# How Smart Can Museums Be? The Role of Cutting-Edge Technologies in Making Modern Museums Smarter

**DOI:** 10.12688/f1000research.156212.1

**Published:** 2025-05-07

**Authors:** Charis Avlonitou, Eirini Papadaki, Androniki Kavoura

**Affiliations:** 1 Department of Business Administration and Tourism, Hellenic Mediterranean University, Heraklion, Greece; 2 Department of Business Administration, University of West Attica, Athens, Attica, Greece

**Keywords:** the smart museum, the 21st-century museum, smart museum's profile

## Abstract

Despite extensive research on technology-mediated visitor experiences, a holistic approach to the smart museum remains underexplored. This paper addresses the gap by examining novel technologies through a cultural lens, particularly their role in enhancing modern museums. We defined the concept and profile of the 21st-century smart museum based on a synthesis of findings from 83 papers and museum reports (2014–2024) on the modern museum, the museum experience, and the integration of innovative technologies. , Of these, 54 articles focused on six main technology categories—Extended Reality (XR), Artificial Intelligence (AI), Internet of Things (IoT), Robots, Fusion, and others (Blockchain, Holography)—were reviewed. From the intersection of museums and technology in the digital era, 15 key characteristics emerged, outlining the ideal smart museum. This paper aims to serve as a framework and guide for cultural managers, museum professionals, and designers in creating and implementing smart museums.

## Introduction

In our rapidly transforming 21st-century society, the image and role of the museum as we knew it until now are gradually changing, thanks, mainly, to the exploitation of the potential of new emerging technologies. The catalytic experience of the COVID-19 pandemic, which dealt a significant blow to museums by threatening their very viability ( UNESCO report 2021), drastically changed the pace and intensity of the digitization of museum collections and the exploitation of the Web 2.0 ( Corona 2021), especially social media (SM), consolidating online marketing strategies and unleashing the enormous potential of “digital engagement” ( Crooke 2020).

While technological developments accelerate at a frenetic pace towards Web 3.0 and 4.0, heralding the completion and expansion of social transformation through symbiotic human-machine interaction, the cultural managers of this traditionally conservative institution are increasingly convinced of the necessity for them to find new forms of “intelligent” or “smart” management, using Information and Communication Technologies (ICT) ( Trunfio et al. 2021). Thus, they hope to optimise museum operation and visitor experience, increase sustainability and competitiveness, and contribute to social welfare and development.

In this context, numerous studies of the last decades and especially after 2017, mostly pilot ones, focus on a variety of new technologies that find application in museums attempting to upgrade them to smarter ones, while others concentrate on the multifarious and constantly evolving types of technology themselves ( Shah, Fathihin and Ghazali 2018, Ch’ng et al. 2019, Lu et al. 2023). However, although there has been extensive research on technology-mediated visitor experience in recent decades, the issue of a holistic approach to the smart museum has attracted only limited attention ( Lu et al. 2023, Pérez 2023).

Thus, it was deemed appropriate and largely necessary to theoretically and holistically explore the concept of the smart museum, the new generation of museums that succeeds the traditional and the digital, in its entire diverse spectrum, viewing it through the cultural lens. Based on the above purpose, we have defined the following main research objectives:

(1) To explore the relationship between the post-war evolution of the museum institution and its new identity.
(2) To define the concept of the smart museum.
(3) To outline the profile of a holistically smart contemporary museum as it emerges through the exploration of both the utilization of cutting-edge technologies and its current identity.

The objectives of our study encompass its key parameters, which also serve as research questions. To address these questions, we explored two main topics: the museum’s transformation over the past 50 years, shaping its current identity, and the adoption of cutting-edge technologies that define its future profile in the 21st century. Specifically, to understand the modern museum’s identity in the third decade of the 21st century, we examined the socio-economic developments that influenced the institution in the post-war era, particularly from the 1980s onward. We also analyzed the digital transformation and integration of new technologies in museum management, through which we could further delineate the concept of the emerging smart museum of the 21st century, tracing its overall profile in its ideal future version.

## Background research

As a constantly evolving social institution and structure, the museum is continually changing, in direct relation to the surrounding reality. A milestone in its development, after the war, was the emergence of New Museology in the 1980s, a time when the focus of interest in modern museum studies began to shift from collections to the public and society, while the museum from object-centered and curator-centered began to become more and more visitor-centered ( Chen, Ho and Ho 2006).

The cultural shift of this era and the emphasis on the study of visual culture through the development of interdisciplinary disciplines such as “visual semiotics” seriously shook the - dominant until the 1960s-1970s – bourgeois view of the museum, according to which museum objects and art objects, par excellence, possessed an intrinsic value, eloquent for a select elite ( Sifaki 2015). At the same time, it marked the transition from the search for inherent universal principles based on the aesthetic concepts of the Enlightenment to the inclusion of aesthetic aspects in the context of cultural functioning and their correlation to power structures ( Cagliostro 2020).

Museums were then seen as representatives of an aesthetic attitude that encouraged inequality, betraying their “real function”, which, as Bourdieu observed, was to enhance “for some the feeling of belonging and for others the feeling of exclusion” ( Bourdieu, Darbel, and Schnapper 1991, 112). Faced with a multi-layered criticism and the “existential crisis” it triggered, cultural managers felt the need to rethink how they manage and promote museum collections, orienting more than ever to all kinds of potential audiences ( Cagliostro 2020).

The transformation of the perception of the museum’s role in society was similarly facilitated by the gradual collapse of the notion of mass culture as a uniformly passive, easily manipulated consumer public. This idea, largely established by prominent figures of the Frankfurt School ( Adorno 2000, Arendt 1994), has increasingly been challenged ( Paschalidis 2002a).

The development and spread of Cultural Studies, particularly through the University of Birmingham and the influence of Marxist sociologists and other intellectuals like Stuart Hall, Raymond Williams, Umberto Eco, and Herbert Gans, played a crucial role in shifting cultural mentality. This movement challenged the notion of passive consumers, emphasised the significance of popular culture, and promoted the view of cultures as dynamic, reconfigurable systems in which cultural consumers can interpret messages in diverse ways ( Williams 1989, Gans 1974, Paschalidis 2002a).

The pressing economic imperatives that followed the reduction of government support for state-subsidised museums from the 1980s onwards, decisively contributed to this shift toward the public ( Chabouri-Ioannidou 2003, 28), which was also in response to demographic and generational change in the society of the time ( Lu et al. 2023). Since then, public museums have increasingly adopted extroversion and the “consumer model” of management by expanding and enriching their activities, competing, thus, with other “leisure industries” ( Desvallées and Mairesse 2014, 22, 28).

In addition, the evolution of educational theories and the transition from a linear-didactic and teacher-centered to a two-way learning model, or in other words, from a positivist-behaviorist to a constructivist approach, were gradually imprinted on the form of the modern museum institution ( Hooper-Greenhill 1999). The constructivist learning model focuses on both the transmitter and the receiver of the message, since knowledge does not arise as a result of the transmission of a pre-existing intrinsic meaning but as a result of a fluid and non-finite “participatory process”, in the construction of which the viewer actively participates (ibid). The so-called cultural approach, which has found appeal and tends to be widely adopted by the postmodern museum, focuses on how interpretation varies according to visitors’ gender, ethnicity, social class, educational level, personal knowledge, and culture ( Hooper-Greenhill 2000).

Furthermore, the recognition of the dominant role culture plays in development – another important socio-economic factor – has influenced the transformation of the museum into a critical space that encourages dialogue around values and questions directly related to the community it represents. After the failure of the economic-centric model of industrialised Western societies and the collapse of the “myth of development” – most noticeable in the 1970s and 1980s – the human community realised that technological and economic development must be in harmony with social welfare and respect nature, the diversity of human existence and the heterogeneity of cultural traditions, values, and identities ( Morin 1998, 534-545, Paschalidis 2002b). This awareness was reinforced by the unprecedented expansion of the very concept of culture, which in 1982 was recognised within the international community as a multifaceted and complex phenomenon, extending from biodiversity and landscape to living experience and all the varied, contradictory and heterogeneous aspects of life ( Mexico City Declaration 1982). Within the same community, a culture-centered approach that places the human community at the center of the development process was adopted and crystallised in a series of normative texts in the early decades of the 21st century, particularly after the catalytic events of 9/11. These documents formulate the framework of a new ethic of safeguarding and promoting intercultural dialogue and sustainable development, as defined based on the UN’s 2030 Agenda for joint social, environmental, and economic action ( United Nations 2015).

As the transformation of the museum institution is reflected in the evolution of its definitions of the period 1946-2022, the most recent of them indicates the new pluralistic, and complex - compared to the traditional - role of the modern museum in society ( Lehmannová 2020). According to it, museum “researches, collects, conserves, interprets and exhibits tangible and intangible heritage”, is “open” to the public, “accessible and inclusive”, fosters “diversity and sustainability”, operates and communicates “ethically, professionally and with the participation of communities, offering varied experiences for education, enjoyment, reflection and knowledge sharing” ( ICOM 2022).

In this context, museum functions combine the traditional functions of acquisition, conservation, and research with management and the more visible function of communication (which includes education-mediation, entertainment, and exhibition). Communication, not traditionally included in the main museum functions, gradually evolved during the late 20th century into the main focus and driving force of its operation ( Desvallées and Mairesse 2014, 21, 60-61). Admittedly, in this new, multifunctional and broad-oriented modern museum, where the complexity of contemporary postmodern society is reflected, the role of technology is catalytic.

In more detail, the digital revolution is the last but far from insignificant socio-economic development that contributed drastically – and still contributes - to shaping the identity of the modern museum. Indeed, the emergence of the new trend of using ICT to promote user interaction, in the late 90s, found full expression in the participatory online cultures of Web 2.0 and especially SM ( Refae 2022). Thus, the cultural recycling of material available on the internet was mixed with people’s daily lives and creative elements, which effortlessly led to a new democratised form of participatory popular culture consumption and production, which included everything as common property and heritage of humanity-audience and educated an audience whose perception was multiplying and expanding relentlessly and feverishly thanks to the internet ( Beer and Burrows 2010, 7, 10-11).

Notwithstanding, the museum sector has indeed been until recently more hesitant and relatively reluctant to follow the digital transformation process compared to other sectors, such as the tourism industry ( Pérez 2023). However, the technological development and innovations of the first decades of the 21st century, such as the emergence of smart mobile phones, developments in computer graphics or software and hardware developments in extended technologies, combined with the aforementioned socio-economic developments and lessons learned since the recent pandemic that has accelerated the digitization processes of the museum, have significantly contributed to the gradual adoption of new technologies by cultural managers ( Margetis et al. 2020, Avlonitou and Papadaki 2024).

Thus, recently, especially in the last decade (with exponential progress from 2017 onwards), many studies have come to light, where researchers propose innovative designs or applications and smart solutions applicable in one or even more museums ( Lu et al. 2023). Scholars investigate “smart learning environments” ( Kasperiuniene and Tandzegolskiene 2020, Pan, Zhang, and Yang 2022), and “intelligent cultural spaces” ( Chianese, Piccialli, and Valente 2015), the utilization of a fusion of technologies towards a specific type of a “smart museum” ( Siountri, Skondras, and Vergados 2019), the digital design of a “smart museum based on AI” ( Wang 2021), IoT-based smart museums ( Mighali et al. 2015, Korzun et al. 2016, Spachos and Plataniotis 2020), cognitive robots for smart museums ( Saggese, Vento, and Vigilante 2019), “smart things” ( Bessaa, Levillain, and Tijus 2020), “smart objects” ( López-Martínez, Carrera, and Iglesias 2020) and “smart showcases” ( Bhattacharya 2019), the “engineering of smart museum ontology” ( Zachila et al. 2021), “smart navigations systems” ( Khan et al. 2021), issues of satisfaction and loyalty in the smart museum ( Zhang and Abd Rahman 2022) and many other options.

Actually, scholars examine aspects of the smart museum mainly in terms of technology and equipment, as an information service, or in terms of their impact on visitor experience and behavior, while even evaluation criteria for smart museums have been proposed exclusively related to the satisfaction of visitors ( Liu and Guo 2023). At the same time, the label of “smart” is currently indiscriminately applied to any integration of new technologies in the museum space, betraying ambiguity and heterogeneity in its use ( Pérez 2023).

We reviewed 83 publications on the smart museum concept through a cultural lens, including relevant museum practices. These publications generally address the integration of new technologies and theoretical issues in modern museums (29 articles), covering six key categories of cutting-edge technologies used in museums: Extended Reality (XR) [24 articles], Artificial Intelligence (AI) [9 articles], Internet of Things (IoT) [7 articles], Robots [7 articles], Fusion [5 articles], and Blockchain and Holography [2 articles]. Of the studies reviewed, ten were published between 2014 and 2016, with the majority published between 2017 and 2024.

Surprisingly, there is a scarcity or lack of holistic models regarding the concept of a smart museum through a cultural lens. Furthermore, there is not a one-size-fits-all definition of the smart museum and the latter is usually integrated into the more general view of intelligent space or simply associated with smart technology. As an exception, Liu and Guo (2023) correlate the smart museum with three aspects, namely protection, management, and service, while Pérez (2023) adopts a more holistic view proposing a model of smart management, which is inspired by and depends on the Spanish model of tourism development SEGITTUR.

According to Pérez’s model for museums, a smart museum is a museum “that makes a transversal incorporation of inclusive strategies and Information and Communication Technologies in all functional levels to obtain an efficient and sustainable management” ( Pérez 2023). The model is namely based on functions related to “the visitor experience”, “conservation and management”, and “marketing and communication strategies”, including Technology, Sustainability, Governance, Accessibility, and Innovation and thus incorporating the five smart axes currently applied to the management of smart destinations as adopted by the above-mentioned tourism management model (ibid).

In another approach, we attempt to examine the contemporary physiognomy of the museum as dictated by recent developments in the museum institution and relate it to the findings of the literature based on the integration of new technologies in museums. Thus, we proceed to fully define the concept of the smart museum by formulating the following definition:

A smart museum is a museum that optimises all its operations and functions through the appropriate use of ICT, staying aligned with the digital age, its mission and the needs of a widened, diverse and inclusive audience, while mobilizing their intelligence, encouraging their interaction with the museum, with each other and with other communities, and focusing on both museum exhibits and on broader social issues.

**Figure 1.**
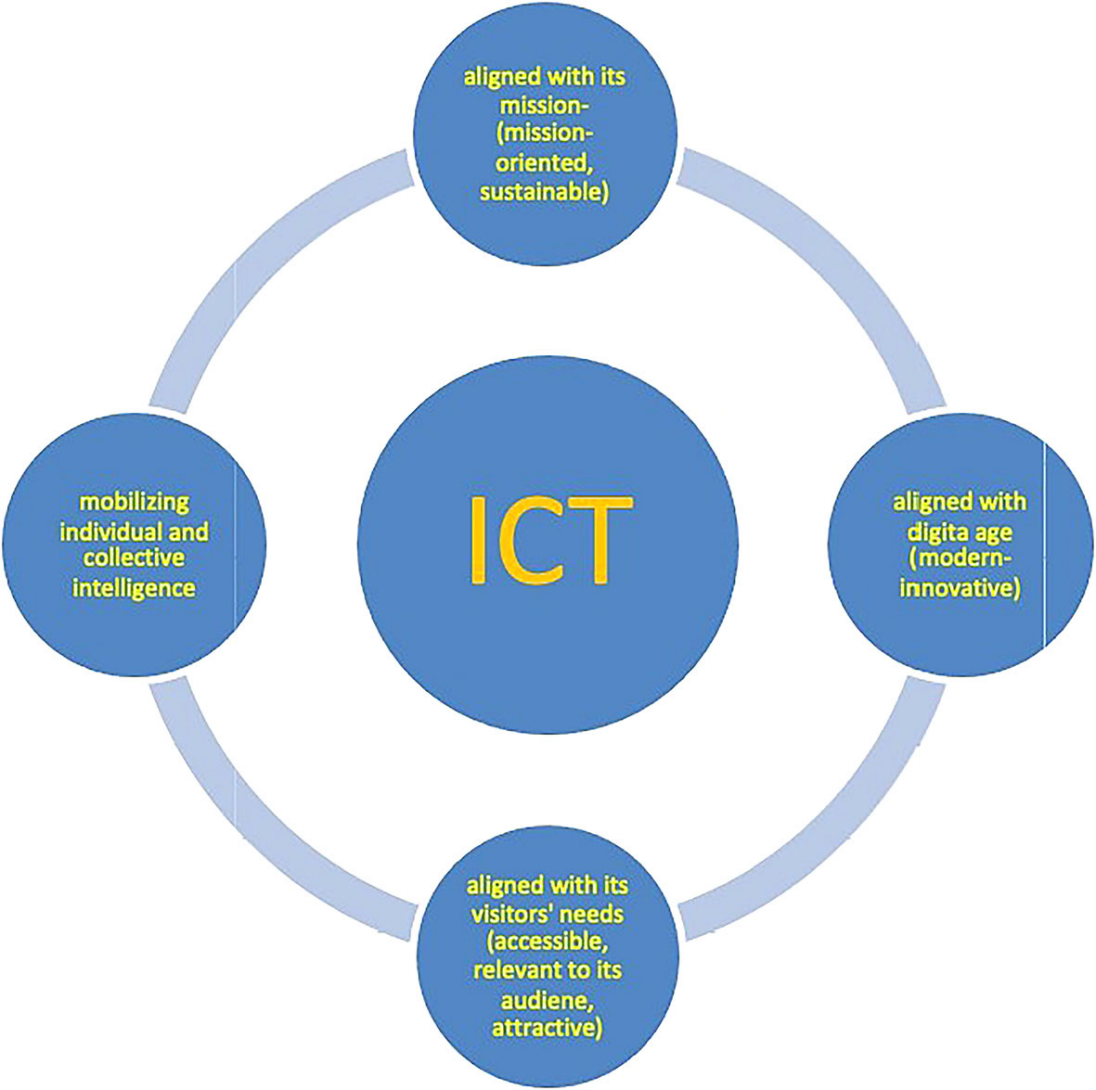
The smart museum diagram

The above definition becomes more readable in the diagram below ( Figure 1), which visualises and distributes the characteristics of the smart museum identity into four categories, the last two of which refer, characteristically, to the function of communication: alignment with museum mission (mission-oriented, sustainable), the digital age (modern-innovative), and the visitors’ needs (accessible, relevant, and attractive to an extensive audience), as well as mobilizing of individual and collective intelligence (of people and local communities). We are going to analyze these features further below.

## Methods

Our investigation was based on an extensive literature review, secondary data classification, and analysis, interpretation, and synthesis methods. The research synthesis method, defined as “a tool for understanding a body of literature and characteristics that enhance or diminish relationships of interest” ( Steingut, Patall, and Fong 2022), was employed to capture the global state of research on the smart museum concept. This method focused on the integration of cutting-edge technologies in contemporary museums and aimed to provide a clear overview of the smart museum’s profile through a cultural lens.

More specifically, the definition of the three main research objectives described above was followed by a comprehensive literature search, involving multiple and complex queries across various available databases, specifically Google Scholar, Scopus, Emerald Insight, ScienceDirect, Springer Link, and ResearchGate. In addition, the MuseWeb database, an annual global conference where advanced research studies and applications of digital practice and innovation in museums are published, was explored and papers relevant to the research topic were gleaned.

The studies were evaluated for their relevance to the predefined research objectives. The material was thematically classified into distinct study groups and subgroups based on qualitative similarities. The collected data were processed, analyzed, and interpreted by inductive and deductive methods with explanatory, descriptive, interpretive, and exploratory purposes.

Papers, sites or documents (83 publications) referring to the utilization of novel technologies and its impact on the physiognomy of the 21st-century museum, were correlated and embedded in a broader theoretical framework, through conceptual principles and approaches such as the theories of “flow”, “value co-creation” and “digital humanism” as well as models of “experience economy” and technology acceptance (8 papers, books or documents, published between 1989 to 2021). Finally, our findings were visualised and distributed in a table with the ultimate goal of clarifying the concept of the smart museum, by outlining the main characteristics that shape its profile. Thus, 15 characteristics and properties were identified and used as the basis of a synthesis depicting the profile of the modern museum in the digital age.

The study focused on both academic research and museum practices of integrating smart solutions in museum environment internationally (Europe, Asia, and America). We studied a broad range of implementations in the contemporary museum while pointing out the obvious benefits to the museum community and inherent challenges. The review followed a general and non-exhaustive/systematic approach and included only English-language literature.

## Findings

Our findings, visualised in Table 1, provide a clear picture of what a smart museum of the future might be. They contribute to helping us understand how the digital transformation of the museum is changing its structure, making it extremely powerful compared to the traditional, i.e. hybrid, modern/innovative, and technologically upgraded, but also the necessity of collaborations and interdisciplinarity to keep up with the digital age.

**Table 1.** The Smart Museum Features Based onLiterature Findings.

Features	Description	Author/s
hybrid	Museums no longer operate solely in the physical environment but are also expanding into the digital space. The visiting experience becomes holistic - before, during and after the physical visit - and ubiquitous.	Black 2018, Ming 2018, Bowen and Giannini 2019, Nisiotis and Alboul 2021, Hutson and Hutson 2023, Calvi & Vermeeren, 2023, Modliński, Fortuna, and Rożnowski 2023.
modern-contemporary innovative	Museums conceptualise themselves as “contemporary institutions” by paving new paths through digital experimentation and innovation. They bridge the past with the future and the intangible with the tangible.	Bowen and Giannini 2019, Scott, Salili-James, and Smith 2023, Recupero et al. 2019.
collaborative/interdisciplinary	The technological requirements and opportunities of the post-pandemic era create a need for collaborations and the integration of new disciplines and specialties within the museum environment.	Fernandes and Casteleiro-Pitrez 2023, Modliński, Fortuna, and Rożnowski 2023, Ludden and Russick 2020.
upgraded	ICT tools enhance the supervision of management operations and processes, making them increasingly automated, interconnected, efficient, and faster. As a result, all museum functions are upgraded.	Ciecko 2017, Summers 2019, Bobasheva, Gandon, and Precioso 2022, Cai, Zhang, and Younghwan 2023, Bhattacharya 2019, Varitimiadis et al. 2020, Frank and Frank 2022, “How Wireless Data Loggers,” n.d. Brondi and Carrozzino 2015, Chianese and Piccialli 2014, Mighali et al. 2015, Gaugne et al. 2022, Mucchi, Milanesi, and Becagli 2022, Khan et al. 2021, Siountri, Skondras, and Vergados 2019
visitor-centered	The smart museum provides personalised guidance and recommendations, and is interactive, participatory and co-creative. Visitors can engage with and even influence the cultural content produced, thereby enriching their experience.	Leue, Jung, and tom Dieck 2014, Khan et al. 2021, Saggese, Vento, and Vigilante 2019, Chen, Ho and Ho 2006, Ch’ng et al. 2019, Iio et al. 2019, Frost, Thomas, and Forbes 2019, Kayukawa et al. 2023, Lupetti, Germak, and Giuliano 2015, Palombini 2017, Black 2018, Bessaa, Levillain, and Tijus 2020, Recupero et al. 2019, Fuentes-Moraleda et al. 2021, Komarac and Došen 2023.
relevant-attractive	The integration of ICT and the innovative nature of the visitor-oriented smart museum foster personal relevance and a sense of belonging for its audiences, particularly younger visitors.	Cerquetti 2016, Recupero et al. 2019, Hughes and Moscardo 2019, Longo and Faraci 2023, Zollo et al. 2021, Manna & Palumbo, 2018, Fernandes and Casteleiro-Pitrez 2023, Zhang and Abd Rahman 2022
provider of immersive experiences	By creating immersive and engaging exhibitions with high-quality content, mediated through seamless and non-intrusive technology, the smart museum experience imprints knowledge indelibly on visitors’ memories.	Csikszentmihalyi 1990, Zhang and Abd Rahman 2022, Pine and Gilmore 2013, London 2020, Komarac and Došen 2023, Longo & Faraci, 2023, Yang 2023, Davis 1989, Leue, Jung, and tom Dieck 2014, Ming 2018, Fernandes & Casteleiro-Pitrez, 2023, Cagliostro 2020.
flexible/up-to-date	To remain attractive and relevant to visitors' interests, the smart museum must stay current, engage with its community, and continuously reconfigure itself.	Khadraoui 2019
educational – entertaining	Museums blend education with recreation to spark curiosity, stimulate critical thinking, and promote self-directed learning. They employ a "hands-on" approach, storytelling, gamification, and other techniques to enhance participation, co-creation, and personal understanding of cultural content.	Trunfio et al. 2021, Marques 2017, Kasperiuniene and Tandzegolskiene 2020, Fernandes and Casteleiro-Pitrez 2023, Pine and Gilmore 2013, Pan, Zhang, and Yang 2022, Modliński, Fortuna, and Rożnowski 2023, Palombini 2017, Black 2018, Harrington 2019, Calvi and Vermeeren 2023, López-Martínez, Carrera, and Iglesias 2020, Katz 2019, “MuseumsQuartier Wien” 2020, Gao and Braud 2023, “Seeing Impressionism” 2019, “ANTEPRIMA La Città Proibita VR” 2016, Rizvic et al. 2022, “ Interactive Holograms” n.d., Longo & Faraci, 2023, Marques 2017, Liestøl 2021, Lee, Park, and Lee 2022
socially engaged and relationally supportive	Smart museums address people’s needs for networking, crowdsourcing, social interaction, and collective engagement. They foster a sense of togetherness, offer a platform for sharing information, inspire and support action, and raise public awareness.	Black 2018, Rahimi 2014, Olaz et al. 2022, Mason 2016, Bowen and Giannini 2019, Refae 2022, Behera 2022, Hourdakis and Ieronimakis 2020, Calvi and Vermeeren 2023, Papadaki 2019, Kyprianidou and Papadaki 2018, “ Culture in Crisis · V&A” n.d.
authentic	Extended technologies could transform the notion of what is considered “authentic” or “real.” Therefore, managing the risk of losing the authenticity and intrinsic value of exhibits is crucial. The originality and authenticity of museum experiences shape the new sensibility of visitors (consumers) in the era of the “experience economy.”	Calvi and Vermeeren 2023 Longo & Faraci, 2023, Ming 2018, Pine and Gilmore 2013.
anthropocentric	Technology is merely a tool to serve people and advance social development. Exaggerations should be avoided, as they can diminish value, disorient museum managers, and cause them to lose focus on the museum's goals.	Bowen and Giannini 2019, Ming 2018, De Angeli and O'Neill 2015, Fernandes and Casteleiro-Pitrez 2023, Chu et al. 2022, Modliński et al., 2023, Kirova 2020, Tassis 2019.
adopter of calculated approach	A calculated approach to museum functions—such as through strategic exhibition design, systematic use of data, assessment tools, and conceptual models for effective technology utilization—is employed by museum policymakers, taking into account the interaction between visitors, technology, and the museum's context and mission.	Modliński, Fortuna, and Rożnowski 2023, Recupero et al. 2019, Ludden and Russick 2020, Neuhofer, Buhalis, and Ladkin 2013, Huang and Rust 2018, YiFei and Othman 2024.
democratizing	Museums as catalysts for dialogue, active participation, social interaction and learning, play a crucial role in democratizing culture and society.	Hourdakis and Ieronimakis 2020, Hutson and Hutson 2023, “V&A Annual Report 2020 to 2021” 2022, “V&A Annual Report 2022 to 2023” 2023.
sustainable	Technology is used to preserve museum collections for the future, facilitate alternative financing (e.g., e-commerce), attract audiences and investment, and contribute to sustainable development by sensitizing the audience to environmental and societal issues and fostering a new mindset and ethics.	Zhang et al. 2022, OECD/ICOM 2019, “V&A Annual Report 2021 to 2022” 2022, “V&A: Sustainability”, n.d., United Nations 2015.

They also demonstrate the positive impact of technology on the visitor experience, as it provides personalization, interactivity, and participation opportunities, and its power to create immersive and engaging worlds that transform the traditional museum into a new, relevant, and engaging one for visitors, especially the youth, provided it stays in touch with their needs and ensures an unobstructed and discreet technological mediation.

The findings of our study also show how the integration of new and emerging technologies leads to a new type of educational-entertainment museum character, capable of facilitating visitors’ critical thinking and personal understanding of cultural heritage (CH) and at the same time socializing their experience and contributing to knowledge sharing and public awareness. Finally, they identify a challenge that technology-mediated experiences in museums can pose, namely the risk of altering or losing the sense of authenticity and neglecting the museum’s objectives, underscoring the need for smart museums to adopt a calculated approach and ensure both their sustainability and their catalytic role in the democratization of culture.

## Result

### Looking to the future of the smart museum: Properties and features

Since the advent of the Internet in 1989, but mainly with the implementation of Web 2.0 and the spread of smartphones in the first decade of the 21st century, digital culture has accelerated rapidly and dominantly. In today’s post-digital age, where physical and digital lives are increasingly merged and interconnected, people are continuously sharing thoughts, images, and knowledge through the Internet, creating a global culture without physical boundaries. As digitization—initially of documents and now of our lives—becomes a central driver of human action, it transforms the way we exist, think, and communicate ( Black 2018, Bowen and Giannini 2019).

As digital culture permeates all social sectors, ICT tools are gradually reshaping museum management, practices, communication strategies, and the way museum exhibitions are designed. By integrating digital technologies at an increasing rate, museums are undergoing a digital transformation, where artificial agents, living organisms and human beings become informational entities and everything is fused into a single space of an informational nature, an ‘infosphere’, that according to Simone, Cerquetti, and La Sala (2021) “reontologises” (i.e., intrinsically redefines) our world.

Considering these tectonic changes and the content of the previous sections, we summarise the 15 key characteristics of the smart museum, aligning them with its definition. These characteristics highlight its unique relationship with the digital age, its mission, the needs of its audience, and its connection to the people and community it serves ( Table 2).

**Table 2.** The Smart Museum Profile: Key Features.

aligned with the digital age	aligned with its mission	aligned with visitors' needs	mobilizing indivicual and collective intelligence
• hybrid • modern-innovative • collaborative-interdisciplinary • upgraded	• authentic • anthropocentric • adopter of calculated approach • democratic • sustainable	• visitor-centered • relevant and attractive to its audience • provider of immersive experiences • flexible and up-to-date	• educational-entertaining • socially connected and relationally supportive

These features—representing the ideal qualities of a holistic smart museum, which emerged through the analysis, processing, and synthesis of our literature findings and were organised into four thematic sections in our description—are further analyzed and illustrated in a summary table ( Table 3). The summary table provides a comprehensive overview of the ideal profile of the smart museum, detailing its main characteristics, indicative methods of implementation and activation in the modern hybrid museum (including means and methods of achievement), and the anticipated benefits of these implementations.

**Table 3.** The Smart Museum Profile: Detailed Description.

Smart museum profile (Key features)			
	Features/properties	Means/methods of achievement	Benefits
1	**aligned with digital age** • hybrid • modern (contemporary)-innovative • collaborative- interdisciplinary • upgraded	• is digitally transformed • is ubiquitous (before, during, and after the visit) • bridges the past with the present through emerging technologies (e.g. XR) • all its functions and operations become more automated and interconnected (e.g. through AI, IoT and Blockchain technologies) • collaborates with IT professionals, cultural organizations, and communities	• expansion of physical boundaries • revitalization of visitor experiences • strengthening of museum competitiveness • attraction of younger visitors • participatory network development • acceleration and efficiency of management
2	**aligned with its mission** • authentic • anthropocentric • adopter of calculated approach • democratic • sustainable	• applies control mechanisms to balance authenticity and technological mediation (e.g. via ethics committees, conducting audience surveys) • maintains a balance between technology (e.g. XR, robots) and strategic planning, regulates ethical issues and serves people, society, and museum goals • applies smart solutions (e.g. by monitoring indicators) to optimize its functions (e.g. reduce its ecological impact) and achieve its objectives • promotes increased accessibility and audience participation for socially excluded or marginalized groups, while fostering diversity and equity • makes its collections accessible to all and preserves them for future generations • uses digital platforms to attract financial and professional capitals or promote alternative financing	• safeguarding and implementation of museum mission • ensuring the leading role of humans in digital transformation • production of added value • contribution to democratization of culture and society • facilitation of museum's sustainability • contribution to environmental, social, and economic development
3	**aligned with visitors' needs** • visitor-centered • relevant and attractive to its audience • provider of immersive experience • flexible and up-to-date	• uses personalization and interactivity techniques to be accessible and comprehensible to all (e.g. via IoT, AI or robots/chatbots) • creates participatory and meaningful experiences/indelible memories (e.g. through “serious games” and XR technologies) • produces a sense of belonging to its audiences (e.g. through digital relationship marketing) • provides high-quality and renewable content via immersive technologies • ensures that the technology is seamless, minimally intrusive and easy to use • remains in touch with reality/visitors' needs (e.g. audience surveys via SM)	• enhancement of visitor experience • production of a state of “flow” and excitement • generation of overall visitor satisfaction • visitor's engagement with CH • integration of museums into everyday life • cultivation of a sense of belonging • support to the museum (e.g. through e-WOM) • retention of existing audiences
4	**mobilizing individual and collective intelligence** • educational-entertaining • socially engaged and relationally supportive	• is experiential (e.g. provides immersive, and participatory experiences through XR, or activates engagement via SM) • uses storytelling and gamification techniques (on site or online) • encourages visitor-generated content (e.g. through SM) • allows tailored content (e.g.through personalized museum guides) • reevaluates hierarchies by abandoning the voice of authority and encouraging public's participation (e.g. via museum sites or SM) • offers individual time management (offers opportunities for “stagnant time”) • uses relationship marketing, SM, digital platforms or conferences to foster dialogue, sharing, social interaction and citizen involvement/participation	• reevaluation of the museum as a reference entity • awakening of curiosity • activation of informal and self-directed learning • stimulation of thought and reflection • promotion of empathy and co-creativity • development of long-term relationships of trust and mutual benefit • strengthening of bonds between museums and the local community • reinforcement of social cohesion • empowerment of collective intelligencefor societal benefit

Thus, the smart museum is outlined as a hybrid, ubiquitous and modern entity that spans space and time, experimenting with and fully adopting smart solutions through both established (e.g., SM) and emerging technologies of the digital era (e.g., XR, IoT, AI, robots, holograms). It remains contemporary, innovative, and upgraded in all its operations—primarily automated, interconnected, and capable of delivering faster services and more efficient management. In this context, it forges collaborations with similar entities and technology consultants, while embracing new disciplines beyond traditional curatorship to acquire skills essential for meeting the modern needs of the post-pandemic era.

At the same time, the smart museum safeguards the authenticity and inherent values of CH from trivialization, offers genuine experiences to its visitors, and promotes digital humanism by balancing the virtual and the real. It preserves its mission and emphasises the human element in the museum experience. Consequently, it fosters the democratization of culture for all individuals while adopting a calculated approach and leveraging smart technological tools and solutions to ensure sustainability and contribute to sustainable development.

As a visitor-centered organization, the modern hybrid and participatory museum employs various techniques and technologies to meet the needs and desires of its visitors, continually reforming to stay viable and relevant. It strives to offer multisensory, immersive experiences and create lasting memories, focusing on the perceived usefulness and ease of use of technological tools while delivering satisfying and engaging CH content.

Simultaneously, the smart museum establishes a participatory culture within its context by encouraging visitor-generated content and co-production, particularly among younger audiences, treating them as “prosumers”—both consumers and producers ( Lee, Park, and Lee 2022). This approach stimulates curiosity, reflection, and empathy, thereby activating both cognitive and emotional skills.

 In the same vein, it involves the audience in an informal and lifelong museum learning process based on personal goals. Similarly, it uses both analogical and digital means to facilitate the transmission of knowledge, interpretation, and collective memory as an “open work” ( Cerquetti 2016) to boost social interaction and strengthen the bonds between museum and local communities, by activating their participation and knowledge.

## Conclusions

The rapid technological developments in computer science that have escalated over the past 20-25 years, along with the deliberate and growing adoption of smart technologies by museum policymakers worldwide, underscore the necessity of studying the smart museum. This paper employs an extensive literature review and key theoretical tools to explore the smart museum concept, addressing three core research questions: its origins, definition, and profile. The study details its characteristics based on the application of emerging technologies in 21st-century museums.

Investigating the first research question revealed that the origins of the smart museum are rooted in the extension of socio-economic developments affecting this century-old institution in the digital age. Key factors include the emergence of New Museology and cultural shifts, reduced state support, the decline of mass culture as a model of passive consumption, the development and adoption of a constructivist, interactive learning model, the recognition of a culture-centric development model, and the digital revolution. These changes are reflected in the museum’s current definition and its evolving institutional role in post-war society.

Secondly, the study identified the variety and fragmentation in the use of the term “smart” within the museum field. Efforts were made to establish a clear and comprehensive definition through a cultural lens, demonstrating that smart technologies are valuable tools for achieving the humanitarian goals of the museum rather than ends in themselves. The smart museum is thus approached holistically, extending not only within the museum through its mission and visitor experience, but also beyond, as it is shaped by the digital age and influences the intelligence of individuals and communities interacting with it.

Finally, tracing the museum of the future, an attempt was made to identify its key characteristics as study parameters and design components of a holistically smart museum, shaped through the utilization of new and emerging technologies. Potential limitations, risks, and future prospects were also addressed.

The smart museum profile was depicted through 15 features and properties, grouped into four general categories that correlate the museum with the digital age, its mission, visitors’ needs, and the activation of individual and collective intelligence. Future case studies that use these characteristics as parameters or design elements will assess the value of this profile and further clarify the concept of the smart museum.

### Ethics and consent

Ethical approval and consent were not required.

## Data Availability

No data are associated with this article.

## References

[ref1] Adorno TW : *Σύνοψη της Πολιτιστικής Βιομηχανίας [Résumé about Cultural Industry]. Translated by Lefteris Anagnostou.* Athens: Alexandria Publications;2000. ( (Original work published 1989).

[ref2] ANTEPRIMA La Città Proibita VR – OCULUS RIFT: MAO. 2016. Accessed July 23, 2024. Reference Source

[ref3] Arendt H : *“Κοινωνία και Κουλτούρα” [Society and Culture]. Η κουλτούρα των μέσων. Μαζική κοινωνία και πολιτιστική βιομηχανία [MediaCulture. Mass Society and the Culture Industry].* Livieratos K Fragoulis D , editors. Athens: Alexandria Publications;1994.

[ref4] Avlonitou C Papadaki E : The Role of Social Media Messages in Cultural Communication: The Case Study of an Instagram Reel. *Online Journal of Communication and Media Technologies.* 2024;14(2):e202415–e202415. 10.30935/ojcmt/14291

[ref5] Beer D Burrows R : Consumption, Prosumption and Participatory Web Cultures. *Journal of Consumer Culture.* 2010;10(1):3–12. 10.1177/1469540509354009

[ref6] Behera B : Museum and Pandemics a Cautionary Tale from History: Impact, Innovations, Learning from Crises. *Anthropology and Ethnology Open Access Journal.* 2022;5(2):1–8. 10.23880/aeoaj-16000193

[ref7] Bessaa H Levillain F Tijus C : The Making of Museum Works as Smart Things. *Understanding Human Behaviour in Complex Systems: Proceedings of the Human Factors and Ergonomics Society Europe Chapter 2019 Annual Conference.* 2020.

[ref8] Bhattacharya I : Review of Smart Showcase: A Gift of Internet of Things (IoT) to Museum. 2019. 10.13140/RG.2.2.19697.43368

[ref9] Black G : Meeting the Audience Challenge in the ‘Age of Participation.’. *Museum Management and Curatorship.* 2018;33(4):302–319. 10.1080/09647775.2018.1469097

[ref10] Bobasheva A Gandon F Precioso F : Learning and Reasoning for Cultural Metadata Quality: Coupling Symbolic AI and Machine Learning over a Semantic Web Knowledge Graph to Support Museum Curators in Improving the Quality of Cultural Metadata and Information Retrieval. *Journal on Computing and Cultural Heritage.* 2022;15:1–23. 10.1145/3485844

[ref11] Bourdieu P Darbel A Schnapper D : *The Love of Art: European Art Museums and Their Public.* Cambridge, UK: Polity Press;1991. Reference Source

[ref12] Bowen JP Giannini T : The Digital Future for Museums. *Museums and Digital Culture.* 2019;551–577. 10.1007/978-3-319-97457-6_28

[ref13] Brondi R Carrozzino M : ARTworks: An Augmented Reality Interface as an Aid for Restoration Professionals. *The Lecture Notes in Computer Science.* 2015;384–398. 10.1007/978-3-319-22888-4_28

[ref14] Cagliostro M : Art of Escape, Magic, and Immersive Storytelling: The Museum as a Limitless Escape Game. MW2020: MuseWeb 2020. 2020. Reference Source

[ref15] Cai PY Zhang K Younghwan P : Application of AI Interactive Device Based on Database Management System in Multidimensional Design of Museum Exhibition Content. *Research Square (Research Square).* 2023 June. 10.21203/rs.3.rs-3074947/v1

[ref16] Calvi L Vermeeren A : *Digitally Enriched Museum Experiences – What Technology Can Do.* Museum Management and Curatorship;2023;1–22. 10.1080/09647775.2023.2235683

[ref17] Cerquetti M : More Is Better! Current Issues and Challenges for Museum Audience Development: A Literature Review. *Journal of Cultural Management Policy.* 2016;6(1):30–43. Reference Source

[ref18] Chabouri-Ioannidou A : Στρατηγική Διαχείρισης των Πολιτιστικών Ιδρυμάτων [Management Strategy of Cultural Institutions]. *Πολιτιστική Πολιτική και Διοίκηση: Πολιτιστική Διαχείριση [Cultural Policy and Management: Cultural Management].* Patras: Hellenic Open University;2003;25–60.

[ref19] Chen HC Ho CK Ho MC : New Communication Model Museums|PDF|Constructivism (Philosophy of Education)|Meme. *Scribd.* 2006. Accessed July 22, 2024. Reference Source

[ref20] Chianese A Piccialli F : Designing a Smart Museum: When Cultural Heritage Joins IoT. *IEEE Xplore.* 2014. September 1, 2014. 10.1109/NGMAST.2014.21

[ref21] Chianese A Piccialli F Valente I : Smart Environments and Cultural Heritage: A Novel Approach to Create Intelligent Cultural Spaces. *Journal of Location Based Services.* 2015;9(3):209–234. 10.1080/17489725.2015.1099752

[ref22] Chu J Xi L Zhang Q : Research on Ethical Issues of Artificial Intelligence in Education. *Lecture Notes in Educational Technology.* 2022;101–108. 10.1007/978-981-19-5967-7_12

[ref23] Ch’ng E Cai S Leow F-T : Adoption and Use of Emerging Cultural Technologies in China’s Museums. *Journal of Cultural Heritage.* 2019;37(May):170–180. 10.1016/j.culher.2018.11.016

[ref24] Ciecko B : Examining the Impact of Artificial Intelligence in Museums – MW17: Museums and the Web 2017. 2017. Accessed July 24, 2024. Reference Source

[ref25] Corona L : Museums and Communication: The Case of the Louvre Museum at the Covid-19 Age. *Humanities and Social Science Research.* 2021;4(1):p15. 10.30560/hssr.v4n1p15

[ref26] Crooke E : Communities, Change and the COVID-19 Crisis. *Museum and Society.* 2020;18(3):305–310. 10.29311/mas.v18i3.3533

[ref27] Csikszentmihalyi M : Flow: The Psychology of Optimal Experience. *ResearchGate.* Harper & Row;1990. January 1990. Reference Source

[ref28] Culture in Crisis · V&A: Victoria and Albert Museum. n.d.Accessed July 22, 2024. Reference Source

[ref29] Davis F : Perceived Usefulness, Perceived Ease of Use, and User Acceptance of Information Technology. *MIS Q.* 1989;13(3):319–340. 10.2307/249008

[ref30] De Angeli D O’Neill E : A Smartphone Headset for Augmented-Reality Experiences in Museums|MW 2015: Museums and the Web 2015. 2015. Accessed July 22, 2024. Reference Source

[ref31] Desvallées A Mairesse F : ΒασικέςΈννοιεςτηςMουσειολογίας *Βασικές Έννοιες της Μουσειολογίας* [Basic Concepts of Museology]. 2014. Reference Source

[ref32] Fernandes N Casteleiro-Pitrez J : Augmented Reality in Portuguese Museums: A Grounded Theory Study on the Museum Professionals’ Perspectives. *Multimodal Technologies and Interaction.* 2023;7(9):87–87. 10.3390/mti7090087

[ref33] Frank SJ Frank AM : Complementing Connoisseurship with Artificial Intelligence. *Curator: The Museum Journal.* 2022;65(4):835–868. 10.1111/cura.12492

[ref34] Frost S Thomas MM Forbes AG : Art I Don’t Like: An Anti-Recommender System for Visual Art – MW19|Boston. 2019. Accessed July 22, 2024. Reference Source

[ref35] Fuentes-Moraleda L Lafuente-Ibañez C Alvarez NF : Willingness to Accept Social Robots in Museums: An Exploratory Factor Analysis according to Visitor Profile. *Library Hi Tech.* 2021;40:894–913. 10.1108/lht-07-2020-0180

[ref36] Gans HJ : *Popular Culture and High Culture.* New York: Basic Books;1974.

[ref37] Gao Z Braud T : VR-Driven Museum Opportunities: Digitized Archives in the Age of the Metaverse. *Artnodes.* 2023; (32). 10.7238/artnodes.v0i32.402462

[ref38] Gaugne R Barreau J-B Lécuyer F : EXtended Reality for Cultural Heritage. *Handbook of Cultural Heritage Analysis.* D’Amico S Venuti V editors. Springer, Cham;2022;1405–1437. 10.1007/978-3-030-60016-7_48 Reference Source

[ref39] Harrington M : Virtual Dioramas inside and Outside Museums with the AR Perpetual Garden. MW19: MuseWeb 2019. 2019. Accessed July 12, 2024. Reference Source

[ref40] *How Wireless Data Loggers Streamline Museum Preservation Monitoring*|Onset’s HOBO Data Loggers:n.d.Accessed July 23, 2024. Reference Source Reference Source

[ref41] Hooper-Greenhill E : Σκέψεις για τη μουσειακή εκπαίδευση και επικοινωνία στη μεταμοντέρνα εποχή [Reflections on Museum Education and Communication in the Postmodern Era]. *Αρχαιολογία και Τέχνες (Archeology and Arts).* 1999;72:47–49. Reference Source

[ref42] Hooper-Greenhill E : Changing Values in the Art Museum: Rethinking Communication and Learning. *International Journal of Heritage Studies.* 2000;6(1):9–31. 10.1080/135272500363715

[ref43] Hourdakis A Ieronimakis J : ‘Exhibiting’ Lifelong Learning in Museums: The Museum of Education/Xeniseum as a Space of Civic Understanding and Social Connectedness. *Academia.* 2020;106–131. 10.26220/aca.3219

[ref44] Huang M-H Rust RT : Artificial Intelligence in Service. *Journal of Service Research.* 2018;21(2):155–172. 10.1177/1094670517752459

[ref45] Hughes K Moscardo G : For Me or Not for Me? Exploring Young Adults’ Museum Representations. *Leisure Sciences.* 2019;41(6):516–534. 10.1080/01490400.2018.1550455

[ref46] Hutson J Hutson P : Museums and the Metaverse: Emerging Technologies to Promote Inclusivity and Engagement. *Application of Modern Trends in Museums.* 2023. 10.5772/intechopen.110044

[ref47] ICOM Approves a New Museum Definition: International Council of Museums. 2022. Accessed July 23, 2024. Reference Source

[ref48] Iio T Satake S Kanda T : Human-like Guide Robot That Proactively Explains Exhibits. *Int. J. Soc. Robot.* 2019;12(2):549–566. 10.1007/s12369-019-00587-y

[ref49] Interactive Holograms: Survivor Stories Experience: Illinois Holocaust Museum. n.d.Accessed July 23, 2024. Reference Source

[ref50] Kasperiuniene J Tandzegolskiene I : Smart Learning Environments in a Contemporary Museum: A Case Study. *Journal of Education Culture and Society.* 2020;11(2):353–375. 10.15503/jecs2020.2.353.375

[ref51] Katz B : The Louvre’s First VR Experience Lets Visitors Get close to the ‘Mona Lisa. *Smithsonian Magazine.* 2019. Accessed July 22, 2024. Reference Source

[ref52] Khadraoui WR : Inclusivity Practices & the Real Role of Technology in Art Museums – MW19|Boston. 2019. 2019. Accessed July 23, 2024. Reference Source

[ref53] Khan MN Rahman HU Faisal M : An IoT-Enabled Information System for Smart Navigation in Museums. *Sensors.* 2021;22(1):312. 10.3390/s22010312 35009853 PMC8749525

[ref54] Kayukawa S Sato D Masayuki M : Enhancing Blind Visitor’s Autonomy in a Science Museum Using an Autonomous Navigation Robot.CHI ‘23: Proceedings of the 2023 CHI Conference on Human Factors in Computing Systems. 2023; 541: 1-14. 10.1145/3544548.3581220

[ref55] Kirova V : *Value Co-Creation and Value Co-Destruction through Interactive Technology in Tourism: The Case of ‘La Cité Du Vin.* Bordeaux, France: Wine Museum;2020. *Current Issues in Tourism*, February, 1–14. 10.1080/13683500.2020.1732883

[ref56] Komarac T Došen ĐO : Understanding Virtual Museum Visits: Generation Z Experiences. *Museum Management and Curatorship.* 2023;39:357–376. October, 1–20. 10.1080/09647775.2023.2269129

[ref57] Korzun D Varfolomeyev A Yalovitsyna S : Semantic Infrastructure of a Smart Museum: Toward Making Cultural Heritage Knowledge Usable and Creatable by Visitors and Professionals. *Personal and Ubiquitous Computing.* 2016;21(2):345–354. 10.1007/s00779-016-0996-7

[ref58] Kyprianidou E Papadaki E : Το ψηφιακό μάρκετινγκ σε μουσεία τέχνης και οργανισμούς παραστατικών τεχνών[ Cultural Organizations’ Digital Marketing: Web 2.0 Applications in Art Museums and Performing Arts Organizations]. *Πολιτιστικές βιομηχανίες και Τεχνοπολιτισμός: Πρακτικές και προκλήσεις [Cultural Industries and Technoculture: Practices and Challenges].* Theodosiou A Papadaki E Athens: Nissos;2018.

[ref59] Lee H-K Park S Lee Y : A Proposal of Virtual Museum Metaverse Content for the MZ Generation. *Digital Creativity.* 2022;33:79–95. 10.1080/14626268.2022.2063903

[ref60] Lehmannová M : 224 YEARS of DEFINING the MUSEUM. 2020. Reference Source

[ref61] Leue MC Jung T Dieck D t : Google Glass Augmented Reality: Generic Learning Outcomes for Art Galleries. *Information and Communication Technologies in Tourism.* 2014;2015:463–476. 10.1007/978-3-319-14343-9_34

[ref62] Liestøl G : Museums, Artefacts and Cultural Heritage Sites. *The Journal of Media Innovations.* 2021;7(1):19–28. 10.5617/jomi.8792

[ref63] Liu S Guo J : Smart Museum and Visitor Satisfaction. *Journal of Autonomous Intelligence.* 2023;7(3). 10.32629/jai.v7i3.1242

[ref64] London D : Fostering Deep Engagement and Enhanced Learning through Wonder, Creativity, and Play. MuseWeb20:MW2020. 2020. Reference Source

[ref65] Longo MC Faraci R : Next-Generation Museum: A Metaverse Journey into the Culture. *Sinergie Italian Journal of Management.* 2023;41(1):147–176. Reference Source

[ref66] López-Martínez A Carrera Á Iglesias CA : Empowering Museum Experiences Applying Gamification Techniques Based on Linked Data and Smart Objects. *Applied Sciences.* 2020;10(16):5419. 10.3390/app10165419

[ref67] Lu SE Moyle B Reid S : Technology and Museum Visitor Experiences: A Four Stage Model of Evolution. *Information Technology & Tourism.* 2023;25:151–174. 10.1007/s40558-023-00252-1

[ref68] Ludden J Russick J : Digital Transformation: It’s a Process and You Can Start Now. MuseWeb20:MW2020. 2020.

[ref69] Lupetti ML Germak C Giuliano L : Robots and Cultural Heritage: New Museum Experiences. *BCS Learning & Development.* 2015. 10.14236/ewic/eva2015.36

[ref70] Manna R Palumbo R : What Makes a Museum Attractive to Young People? Evidence from Italy. *International Journal of Tourism Research.* 2018;20(4):508–517. 10.1002/jtr.2200

[ref71] Margetis G Apostolakis KC Ntoa S : X-Reality Museums: Unifying the Virtual and Real World towards Realistic Virtual Museums. *Applied Sciences.* 2020;11(1):338. 10.3390/app11010338

[ref72] Marques D : *The Visitor Experience Using Augmented Reality on Mobile Devices in Museum Exhibitions.* FEUP - Faculdade de Engenharia da Universidade do Porto;2017. Thesis. Reference Source

[ref73] Mason M : The MIT Museum Glassware Prototype. *Journal on Computing and Cultural Heritage.* 2016;9(3):1–28. 10.1145/2872278

[ref74] Mexico City Declaration on Cultural Policies: Mexico City. 1982. Unesco. Reference Source

[ref75] Mighali V Del Fiore G Patrono L : Innovative IoT-Aware Services for a Smart Museum. WWW ‘15 Companion: Proceedings of the 24th International Conference on World Wide Web. 2015; 547-550 10.1145/2740908.2744711

[ref76] Ming X : Augmented Reality (AR) in Art Museums: Reconfiguring and Mediating the Museum Dynamics. *Essay.utwente.nl.* 2018. June 29, 2018. Reference Source

[ref77] Modliński A Fortuna P Rożnowski B : Robots Onboard? Investigating What Individual Predispositions and Attitudes Influence the Reactions of Museums’ Employees towards the Adoption of Social Robots. *Museum Management and Curatorship.* 2023;39:457–481. 10.1080/09647775.2023.2235678

[ref79] Morin E : *Κοινωνιολογία [Sociology]. Translated by* Dimitris Dimoulas . Athens: Ekdosis tou eikostou protou;1998.

[ref80] Mucchi L Milanesi M Becagli C : Blockchain Technologies for Museum Management. The Case of the Loan of Cultural Objects. *Current Issues in Tourism.* 2022;25(18):3042–3056. 10.1080/13683500.2022.2050358

[ref81] MuseumsQuartier Wien as an Interactive Work of Art: B2B Austria. 2020. Accessed July 22, 2024. Reference Source

[ref82] Neuhofer B Buhalis D Ladkin A :2013 Experiences, Co-Creation and Technology: A Conceptual Approach to Enhance Tourism Experiences. Reference Source

[ref83] Nisiotis L Alboul L : Initial Evaluation of an Intelligent Virtual Museum Prototype Powered by AI, XR and Robots. De Paolis LT Arpaia P Bourdot P , editors. *Augmented Reality, Virtual Reality, and Computer Graphics. AVR 2021. Lecture Notes in Computer Science.* vol 12980: Springer, Cham;2021; pp.290–305. 10.1007/978-3-030-87595-4_21

[ref84] OECD/ICOM: Culture and Local Development: Maximising the Impact: A Guide for Local Governments, Communities and Museums. 2019 September. 10.1787/9a855be5-en Reference Source

[ref85] Olaz X Garcia R Ortiz A : An Interdisciplinary Design of an Interactive Cultural Heritage Visit for In-Situ, Mixed Reality and Affective Experiences. *Multimodal Technologies and Interaction.* 2022;6(7):59. 10.3390/mti6070059

[ref86] Palombini A : Storytelling and Telling History. Towards a Grammar of Narratives for Cultural Heritage Dissemination in the Digital Era. *Journal of Cultural Heritage.* 2017;24(March):134–139. 10.1016/j.culher.2016.10.017

[ref87] Pan R Zhang L Yang J : A Systematic Review of Smart Learning Environments. *Lecture Notes in Educational Technology.* 2022;11–20. 10.1007/978-981-19-5967-7_3

[ref88] Papadaki E : The Semiotics of Cultural Organisations’ On-Line Branding: The Examples of the Metropolitan Opera of New York and the National Opera of Greece. *Semiotics and Visual Communication III: Branded. The Semiotics of Branding in Culture and Context.* Zantides E , editor. Newcastle: Cambridge Scholars Publishing;2019; pp.426–49.

[ref89] Paschalidis G : Μαζική Κουλτούρα και Υψηλή Τέχνη [Mass Culture and High Art]. *Οι διαστάσεις των πολιτιστικών φαινομένων [The Dimensions of Cultural Phenomena].* Paschalidis G Chabouri-Ioannidou A , editors. Patras: Hellenic Open University;2002a; pp.87–151.

[ref90] Paschalidis G : Η Συμβολή του Πολιτισμού στην Κοινωνική και Οικονομική Ανάπτυξη [The Contribution of Culture to Social and Economic Development]. *Οι διαστάσεις των πολιτιστικών φαινομένων [The Dimensions of Cultural Phenomena].* Paschalidis G Chabouri-Ioannidou A , editors. Patras: Hellenic Open University;2002b; pp.221–243.

[ref91] Pérez A : Smart Museums. Definition and Presentation of a Smart Management Model for Museums. *Tourism and Heritage Journal.* 2023;4(January):126–139. 10.1344/thj.2022.4.8

[ref92] Pine BJ Gilmore JH : The Experience Economy: Past, Present and Future. *Handbook on the Experience Economy.* 2013;21–44. 10.4337/9781781004227.00007

[ref93] Rahimi BF : A Model for Sociocultural Interactions in Museums. *Museum Management and Curatorship.* 2014;29(2):174–187. 10.1080/09647775.2014.888821

[ref94] Recupero A Talamo A Triberti S : Bridging Museum Mission to Visitors’ Experience: Activity, Meanings, Interactions, Technology. *Front. Psychol.* 2019;10(September). 10.3389/fpsyg.2019.02092 31551900 PMC6746986

[ref95] Refae S : Potential of Adapting Smart Cultural Model Related to Contemporary Art: The Case of Jeddah Open-Air Sculpture Museum. *Civil Engineering and Architecture.* 2022;10(3A):86–92. 10.13189/cea.2022.101311

[ref96] Rizvic S Mijatovic B Boskovic D : Workflow of Extended Reality Applications for Museum Exhibitions. *IEEE Xplore.* 2022. August 1, 2022. 10.1109/BalkanCom55633.2022.9900866

[ref97] Saggese A Vento M Vigilante V : MIVIABot: A Cognitive Robot for Smart Museum. *Lect. Notes Comput. Sci.* 2019;15–25. 10.1007/978-3-030-29888-3_2

[ref98] Seeing Impressionism in 3D: Art Gallery of Ontario. 2019. Accessed July 22, 2024. Reference Source

[ref99] Scott B Salili-James A Smith V : Robot-In-The-Loop: Prototyping Robotic Digitisation at the Natural History Museum. *Biodiversity Information Science and Standards.* 2023;7(September). 10.3897/biss.7.112947

[ref78] Shah MN Fathihin N Ghazali M : A Systematic Review on Digital Technology for Enhancing User Experience in Museums. *Communications in Computer and Information Science.* 2018;886:35–46. 10.1007/978-981-13-1628-9_4

[ref100] Sifaki E : Οπτική Eπικοινωνία και Tέχνες: Ένα Παράδειγμα Ανάλυσης Οπτικού Σχεδιασμού [Visual Communication and the Arts: An Example of Visual Design Analysis]. *Βίωμα και Bασισμένες στην Tέχνη Ποιοτικές Μέθοδοι Έρευνας [Experience and Art-Based Qualitative Research Methods], edited by Marios Pourkos.* Athens: Nisides;2015.

[ref101] Simone C Cerquetti M La Sala A : Museums in the Infosphere: Reshaping Value Creation. *Museum Management and Curatorship.* 2021;36(4):322–341. 10.1080/09647775.2021.1914140

[ref102] Siountri K Skondras E Vergados DD : Towards a Smart Museum Using BIM, IoT, Blockchain and Advanced Digital Technologies. *Proceedings of the 3rd International Conference on Vision, Image and Signal Processing.* 2019. August. 10.1145/3387168.3387196

[ref103] Spachos P Plataniotis KN : BLE Beacons for Indoor Positioning at an Interactive IoT-Based Smart Museum. *IEEE Syst. J.* 2020;14:3483–3493. 10.1109/jsyst.2020.2969088

[ref104] Steingut RR Patall EA Fong CJ : Research Synthesis Methods. *Res. Synth. Methods.* 2022. 10.4324/9781138609877-ree55-1

[ref105] Summers K : Magical Machinery? What AI Can Do for Museums. *American Alliance of Museums.* 2019. May 3, 2019. Reference Source

[ref106] Tassis T : *Ψηφιακός Ανθρωπισμός: Εικονιστικό Υποκείμενο και Τεχνητή Νοημοσύνη [Digital Humanism: Iconistic Subject and Artificial Intelligence].* Athens: Armos Publications;2019.

[ref107] Trunfio M Lucia MD Campana S : Innovating the Cultural Heritage Museum Service Model through Virtual Reality and Augmented Reality: The Effects on the Overall Visitor Experience and Satisfaction. *Journal of Cultural Heritage.* 2021;17(1):1–19. 10.1080/1743873x.2020.1850742

[ref108] UNESCO report: Museums around the World in the Face of COVID-19:2021. Reference Source

[ref109] United Nations: Refworld|Transforming Our World: The 2030 Agenda for Sustainable Development. *Refworld.* 2015; 2015. Reference Source

[ref110] Varitimiadis S Kotis K Skamagis A : Towards Implementing an AI Chatbot Platform for Museums. *International Conference on Cultural Informatics, Communication & Media Studies.* 2020;1(1). 10.12681/cicms.2732

[ref111] V&Α · Sustainability: Victoria and Albert Museum. n.d. Reference Source

[ref112] Victoria and Albert Museum Annual Report and Accounts 2020 to 2021: GOV.UK. 2022. Accessed July 22, 2024. Reference Source

[ref113] Victoria and Albert Museum Annual Report and Accounts 2021-2022:2022. Reference Source

[ref114] Victoria and Albert Museum Annual Report and Accounts 2022-2023:2023. Reference Source

[ref115] Wang B : Digital Design of Smart Museum Based on Artificial Intelligence. Edited by Sang-Bing Tsai. *Mobile Information Systems.* 2021;2021(December):1–13. 10.1155/2021/4894131

[ref116] Williams R : *Resources of Hope: Culture, Democracy, Socialism.* London: Verso;1989.

[ref117] Yang S : Storytelling and User Experience in the Cultural Metaverse. *Heliyon.* 2023;9(4):e14759. 10.1016/j.heliyon.2023.e14759 37035365 PMC10073831

[ref118] YiFei L Othman MK : Investigating the Behavioural Intentions of Museum Visitors towards VR: A Systematic Literature Review. *Computers in Human Behavior.* 2024;155:108167–108167. 10.1016/j.chb.2024.108167

[ref119] Zachila K Kotis K Paparidis E : Facilitating Semantic Interoperability of Trustworthy IoT Entities in Cultural Spaces: The Smart Museum Ontology. *IoT.* 2021;2(4):741–760. 10.3390/iot2040037

[ref120] Zhang R(R) Rahman AA : Dive in the Flow Experience: Millennials’ Tech-Savvy, Satisfaction and Loyalty in the Smart Museum. *Current Issues in Tourism.* 2022;25(22):3694–3708. 10.1080/13683500.2022.2070459

[ref121] Zhang X Yang D Yow CH : Metaverse for Cultural Heritages. *Electronics.* 2022;11(22):3730. 10.3390/electronics11223730

[ref122] Zollo L Rialti R Marrucci A : How Do Museums Foster Loyalty in Tech-Savvy Visitors? The Role of Social Media and Digital Experience. *Current Issues in Tourism.* 2021;25(18):2991–3008. 10.1080/13683500.2021.1896487

