# How Smart Can Museums Be? The Role of Cutting-Edge Technologies in Making Modern Museums Smarter

**DOI:** 10.12688/f1000research.156212.2

**Published:** 2025-11-26

**Authors:** Charis Avlonitou, Eirini Papadaki, Androniki Kavoura

**Affiliations:** 1 Department of Business Administration and Tourism, Hellenic Mediterranean University, Heraklion, Greece; 2 Department of Business Administration, University of West Attica, Athens, Attica, Greece

**Keywords:** the smart museum, the 21st-century museum, smart museum's profile

## Abstract

Despite extensive research on technology-mediated visitor experiences, a holistic approach to the smart museum remains underexplored. This paper addresses the gap by examining the intersection of museums and digital technologies through a cultural lens, with a focus on their role in redefining the museum experience. The concept and profile of the 21st-century smart museum were shaped through a synthesis of findings from 143 scholarly and institutional publications, combining 118 research outputs (2012–2025) across seven technological categories —Extended Reality (XR), Artificial Intelligence (AI), Internet of Things (IoT), Robotics, Fusion, Blockchain, and general Digital Technologies— with a complementary body of theoretical works (1989–2025) that strengthen its conceptual and cultural foundations. From this synthesis, 15 key attributes emerged, outlining the ideal smart museum model. Anchored in theoretical and institutional frameworks, these attributes conceptualize the smart museum as a culturally embedded, technologically enhanced, and human-centered institution. The paper provides a conceptual framework and strategic guide for cultural managers, museum professionals, and designers developing smart museum practices in the digital era.

## Introduction

In our rapidly transforming 21st-century society, the image and role of the museum as we knew it until now are gradually changing, thanks, mainly, to the exploitation of the potential of new emerging technologies. The catalytic experience of the COVID-19 pandemic, which dealt a significant blow to museums by threatening their very viability ( UNESCO report 2021), drastically changed the pace and intensity of the digitization of museum collections and the exploitation of the Web 2.0 ( Corona 2021), especially social media (SM), consolidating online marketing strategies and unleashing the enormous “potential of digital engagement” ( Crooke 2020).

While technological developments accelerate at a frenetic pace towards Web 3.0 and 4.0, heralding the completion and expansion of social transformation through symbiotic human-machine interaction, the cultural managers of this traditionally conservative institution are increasingly convinced of the necessity for them to find new forms of “intelligent” or “smart” management, using Information and Communication Technologies (ICT) ( Trunfio et al. 2021). Thus, they hope to optimise museum operation and visitor experience, increase sustainability and competitiveness, and contribute to social welfare and development.

In this context, numerous studies of the last decades and especially after 2017, mostly pilot ones, focus on a variety of new technologies that find application in museums attempting to upgrade them to smarter ones, while others concentrate on the multifarious and constantly evolving types of technology themselves ( Shah, Fathihin and Ghazali 2018, Ch’ng et al. 2019, Lu et al. 2023). However, although there has been extensive research on technology-mediated visitor experience in recent decades, the issue of a holistic approach to the smart museum has attracted only limited attention ( Lu et al. 2023, Pérez 2023).

Thus, it was deemed appropriate and largely necessary to theoretically and holistically explore the concept of the smart museum, the new generation of museums that succeeds the traditional and the digital, in its entire diverse spectrum, viewing it through the cultural lens. Based on the above purpose, we have defined the following main research objectives:

(1) To explore the relationship between the post-war evolution of the museum institution and its new identity.
(2) To define the concept of the smart museum.
(3) To outline the profile of a holistically smart contemporary museum as it emerges through the exploration of both the utilization of cutting-edge technologies and its current identity.

The objectives of our study encompass its key parameters, which also serve as research questions. To address these questions, we explored two main topics: the museum’s transformation over the past 50 years, shaping its current identity, and the adoption of cutting-edge technologies that define its future profile in the 21st century. Specifically, to understand the modern museum’s identity in the third decade of the 21st century, we examined the socio-economic developments that influenced the institution in the post-war era, particularly from the 1980s onward. We also analyzed the digital transformation and integration of new technologies in museum management, through which we could further delineate the concept of the emerging smart museum of the 21st century, tracing its overall profile in its ideal future version.

## Background research

As a constantly evolving social institution and structure, the museum is continually changing, in direct relation to the surrounding reality. A milestone in its development, after the war, was the emergence of New Museology in the 1980s, a time when the focus of interest in modern museum studies began to shift from collections to the public and society, while the museum from object-centered and curator-centered began to become more and more visitor-centered ( Chen, Ho and Ho 2006).

The cultural shift of this era and the emphasis on the study of visual culture through the development of interdisciplinary disciplines such as “visual semiotics” seriously shook the - dominant until the 1960s-1970s – bourgeois view of the museum, according to which museum objects and art objects, par excellence, possessed an intrinsic value, eloquent for a select elite ( Sifaki 2015). At the same time, it marked the transition from the search for inherent universal principles based on the aesthetic concepts of the Enlightenment to the inclusion of aesthetic aspects in the context of cultural functioning and their correlation to power structures ( Cagliostro 2020).

Museums were then seen as representatives of an aesthetic attitude that encouraged inequality, betraying their “real function”, which, as Bourdieu observed, was to enhance “for some the feeling of belonging and for others the feeling of exclusion” ( Bourdieu, Darbel, and Schnapper 1991, 112). Faced with a multi-layered criticism and the “existential crisis” it triggered, cultural managers felt the need to rethink how they manage and promote museum collections, orienting more than ever to all kinds of potential audiences ( Cagliostro 2020).

The transformation of the perception of the museum’s role in society was similarly facilitated by the gradual collapse of the notion of mass culture as a uniformly passive, easily manipulated consumer public. This idea, largely established by prominent figures of the Frankfurt School ( Adorno 2000, Arendt 1994), has increasingly been challenged ( Paschalidis 2002a).

The development and spread of Cultural Studies, particularly through the University of Birmingham and the influence of Marxist sociologists and other intellectuals like Stuart Hall, Raymond Williams, Umberto Eco, and Herbert Gans, played a crucial role in shifting cultural mentality. This movement challenged the notion of passive consumers, emphasised the significance of popular culture, and promoted the view of cultures as dynamic, reconfigurable systems in which cultural consumers can interpret messages in diverse ways ( Williams 1989, Gans 1974, Paschalidis 2002a).

The pressing economic imperatives that followed the reduction of government support for state-subsidised museums from the 1980s onwards, decisively contributed to this shift toward the public ( Chabouri-Ioannidou 2003, 28), which was also in response to demographic and generational change in the society of the time ( Lu et al. 2023). Since then, public museums have increasingly adopted extroversion and the “consumer model” of management by expanding and enriching their activities, competing, thus, with other “leisure industries” ( Desvallées and Mairesse 2014, 22, 28).

In addition, the evolution of educational theories and the transition from a linear-didactic and teacher-centered to a two-way learning model, or in other words, from a positivist-behaviorist to a constructivist approach, were gradually imprinted on the form of the modern museum institution ( Hooper-Greenhill 1999). The constructivist learning model focuses on both the transmitter and the receiver of the message, since knowledge does not arise as a result of the transmission of a pre-existing intrinsic meaning but as a result of a fluid and non-finite “participatory process”, in the construction of which the viewer actively participates (ibid). The so-called cultural approach, which has found appeal and tends to be widely adopted by the postmodern museum, focuses on how interpretation varies according to visitors’ gender, ethnicity, social class, educational level, personal knowledge, and culture ( Hooper-Greenhill 2000).

Furthermore, the recognition of the dominant role culture plays in development – another important socio-economic factor – has influenced the transformation of the museum into a critical space that encourages dialogue around values and questions directly related to the community it represents. After the failure of the economic-centric model of industrialised Western societies and the collapse of the “myth of development” – most noticeable in the 1970s and 1980s – the human community realised that technological and economic development must be in harmony with social welfare and respect nature, the diversity of human existence and the heterogeneity of cultural traditions, values, and identities ( Morin 1998, 534-545, Paschalidis 2002b). This awareness was reinforced by the unprecedented expansion of the very concept of culture, which in 1982 was recognised within the international community as a multifaceted and complex phenomenon, extending from biodiversity and landscape to living experience and all the varied, contradictory and heterogeneous aspects of life ( Mexico City Declaration 1982). Within the same community, a culture-centered approach that places the human community at the center of the development process was adopted and crystallised in a series of normative texts in the early decades of the 21st century, particularly after the catalytic events of 9/11. These documents formulate the framework of a new ethic of safeguarding and promoting intercultural dialogue and sustainable development, as defined based on the UN’s 2030 Agenda for joint social, environmental, and economic action ( United Nations 2015).

As the transformation of the museum institution is reflected in the evolution of its definitions of the period 1946-2022, the most recent of them indicates the new pluralistic, and complex - compared to the traditional - role of the modern museum in society ( Lehmannová 2020). According to it, museum “researches, collects, conserves, interprets and exhibits tangible and intangible heritage”, is “open” to the public, “accessible and inclusive”, fosters “diversity and sustainability”, operates and communicates “ethically, professionally and with the participation of communities, offering varied experiences for education, enjoyment, reflection and knowledge sharing” ( ICOM 2022).

In this context, museum functions combine the traditional functions of acquisition, conservation, and research with management and the more visible function of communication (which includes education-mediation, entertainment, and exhibition). Communication, not traditionally included in the main museum functions, gradually evolved during the late 20th century into the main focus and driving force of its operation ( Desvallées and Mairesse 2014, 21, 60-61). Admittedly, in this new, multifunctional and broad-oriented modern museum, where the complexity of contemporary postmodern society is reflected, the role of technology is catalytic.

In more detail, the digital revolution is the last but far from insignificant socio-economic development that contributed drastically – and still contributes - to shaping the identity of the modern museum. Indeed, the emergence of the new trend of using ICT to promote user interaction, in the late 90s, found full expression in the participatory online cultures of Web 2.0 and especially SM ( Refae 2022). Thus, the cultural recycling of material available on the internet was mixed with people’s daily lives and creative elements, which effortlessly led to a new democratised form of participatory popular culture consumption and production, which included everything as common property and heritage of humanity-audience and educated an audience whose perception was multiplying and expanding relentlessly and feverishly thanks to the internet ( Beer and Burrows 2010, 7, 10-11).

Notwithstanding, the museum sector has indeed been until recently more hesitant and relatively reluctant to follow the digital transformation process compared to other sectors, such as the tourism industry ( Pérez 2023). However, the technological development and innovations of the first decades of the 21st century, such as the emergence of smart mobile phones, developments in computer graphics or software and hardware developments in extended technologies, combined with the aforementioned socio-economic developments and lessons learned since the recent pandemic that has accelerated the digitization processes of the museum, have significantly contributed to the gradual adoption of new technologies by cultural managers ( Margetis et al. 2020, Avlonitou and Papadaki 2024a).

Thus, recently, especially in the last decade (with exponential progress from 2017 onwards), many studies have come to light, where researchers propose innovative designs or applications and smart solutions applicable in one or even more museums ( Lu et al. 2023). Scholars investigate “smart learning environments” ( Kasperiuniene and Tandzegolskiene 2020, Pan, Zhang, and Yang 2022), and “intelligent cultural spaces” ( Chianese, Piccialli, and Valente 2015), the utilization of a fusion of technologies towards a specific type of a “smart museum” ( Siountri, Skondras, and Vergados 2019), the digital design of a “smart museum based on AI” ( Wang 2021), IoT-based smart museums ( Mighali et al. 2015, Korzun et al. 2016, Spachos and Plataniotis 2020), cognitive robots for smart museums ( Saggese, Vento, and Vigilante 2019), “smart things” ( Bessaa, Levillain, and Tijus 2020), “smart objects” ( López-Martínez, Carrera, and Iglesias 2020) and “smart showcases” ( Bhattacharya 2019), the “engineering of smart museum ontology” ( Zachila et al. 2021), “smart navigations systems” ( Khan et al. 2021), issues of satisfaction and loyalty in the smart museum ( Zhang and Abd Rahman 2022) and many other options.

Actually, scholars examine aspects of the smart museum mainly in terms of technology and equipment, as an information service, or in terms of their impact on visitor experience and behavior, while even evaluation criteria for smart museums have been proposed exclusively related to the satisfaction of visitors ( Liu and Guo 2023). At the same time, the label of “smart” is currently indiscriminately applied to any integration of new technologies in the museum space, betraying ambiguity and heterogeneity in its use ( Pérez 2023).

Surprisingly, there is a scarcity or lack of holistic models regarding the concept of a smart museum through a cultural lens. Furthermore, there is not a one-size-fits-all definition of the smart museum and the latter is usually integrated into the more general view of intelligent space or simply associated with smart technology. As an exception, Liu and Guo (2023) correlate the smart museum with three aspects, namely protection, management, and service, while Pérez (2023) adopts a more holistic view proposing a model of smart management, which is inspired by and depends on the Spanish model of tourism development SEGITTUR.

According to Pérez’s model for museums, a smart museum is a museum “that makes a transversal incorporation of inclusive strategies and Information and Communication Technologies in all functional levels to obtain an efficient and sustainable management” ( Pérez 2023). The model is namely based on functions related to “the visitor experience”, “conservation and management”, and “marketing and communication strategies”, including Technology, Sustainability, Governance, Accessibility, and Innovation and thus incorporating the five smart axes currently applied to the management of smart destinations as adopted by the above-mentioned tourism management model (ibid).

Adopting a new approach, this article examines the contemporary physiognomy of the museum as shaped by recent institutional developments and an extended literature review on the integration of emerging technologies in modern museology. Within this framework, the concept of the smart museum is articulated through the following definition:

A smart museum is a museum that optimises all its operations and functions through the appropriate use of digital technologies, staying aligned with the digital age, its mission and the needs of a widened, diverse and inclusive audience, while mobilizing their intelligence, encouraging their interaction with the museum, with each other and with other communities, and focusing on both museum exhibits and on broader social issues.

**Figure 1.**
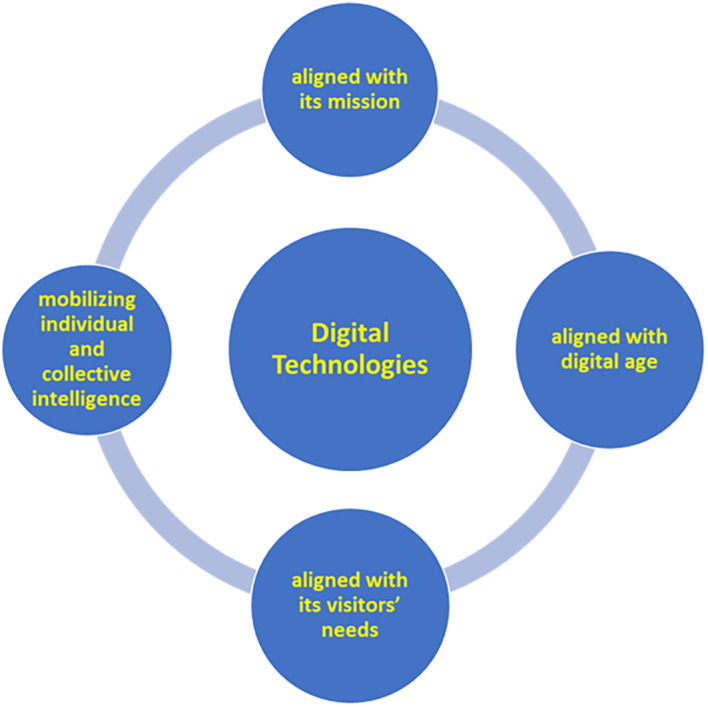
Smart museum concept definition diagram.

The above definition becomes more readable in the diagram below ( Figure 1), which visualises and distributes the attributes of the smart museum identity into four foundational categories or pillars, the last two of which refer, characteristically, to the function of communication: alignment with museum mission (mission-oriented, sustainable), the digital age (modern-innovative), and the visitors’ needs (accessible, relevant, and attractive to an extensive audience), as well as mobilizing of individual and collective intelligence (of people and local communities). The attributes comprising these four pillars (mission-driven, technological, visitor-centered, socio-cultural) will be analyzed in detail below.

## Methods

Our investigation was based on an extensive literature review, secondary data classification, and analysis, interpretation, and synthesis methods. The research synthesis method, defined as “a tool for understanding a body of literature and characteristics that enhance or diminish relationships of interest” ( Steingut, Patall, and Fong 2022), was employed to capture the global state of research on the smart museum concept. This method focused on the integration of cutting-edge technologies in contemporary museums and aimed to provide a clear overview of the smart museum’s profile through a cultural lens.

More specifically, the definition of the three main research objectives described above was followed by a comprehensive literature search, involving multiple and complex queries across various available databases, specifically Google Scholar, Scopus, Emerald Insight, ScienceDirect, Springer Link, and ResearchGate. In addition, the MuseWeb database, an annual global conference where advanced research studies and applications of digital practice and innovation in museums are published, was explored and papers relevant to the research topic were gleaned. References from the international EVA (Electronic Visualisation and the Arts) conferences were also considered.

The collected materials were systematically organized into study groups by type and thematic focus. These comprised publications on specific technologies, theoretical and analytical studies, and institutional documents providing conceptual frameworks for analyzing the contemporary museum as a 21st-century smart museum—integrating technological, organizational, and experiential dimensions. Technology-oriented studies were further subdivided by focus area, including Artificial Intelligence (AI), the Internet of Things (IoT), Extended Reality (XR), Blockchain, Fusion Technologies, Digital Technologies (spanning emerging and social media forms), and Analog Modes of Intervention. All data were systematically processed and analyzed through a combination of inductive and deductive approaches, serving explanatory, descriptive, and exploratory aims. Comparative methods were employed to identify recurrent themes, conceptual trends, and evolving patterns in the discourse on the smart museum.

The study focused on both academic research, institutional documents, and museum practices related to the integration of smart solutions in museum environments worldwide (Europe, Asia, America). A general, non-exhaustive, and non-systematic approach was adopted to capture the diversity of practices and perspectives shaping the field. A broad spectrum of implementations within contemporary museums was examined, emphasizing both the tangible benefits for the museum community and the inherent challenges associated with technological transformation.

Within this framework, academic literature—including 120 peer-reviewed journal articles, conference papers, and peer-reviewed or editorially reviewed academic monographs or book chapters—constituted the core body of research, primarily examining the adoption of novel technologies and their impact on the physiognomy of the 21st-century museum. These were complemented by 23 additional institutional and professional sources issued by internationally recognized organizations, including major museum websites showcasing current applications of emerging technologies not yet represented in academic publications, annual reports, policy papers, and non-regulatory frameworks from international and intergovernmental bodies such as ICOM, UNESCO, OECD, and the European Union.

These complementary materials served to contextualize the evolution of digital technologies within broader ethical, cultural, and strategic frameworks, particularly in relation to challenges posed by emerging technologies such as AI. The study of the collected material—especially case studies involving emerging technologies—was embedded within a broader theoretical framework informed by conceptual models of the “experience economy” and by theories of “flow,” “value co-creation,” and “digital humanism.” Theoretical and empirical references spanning the period from 1989 to 2025 thus provided a comprehensive basis for interpreting the ongoing transformation of the museum in the digital and smart era.

At the analytical level, the main categories of sources employed were identified and classified. The analysis also examined the frequency of references to specific technologies, the distribution of focus within the XR spectrum, and the chronological and geographical distribution of peer-reviewed publications (journal articles and conference papers) by year and country. The results were visualized through corresponding charts, including pie, line, and bar/column graphs.

Two additional heatmaps were generated to elucidate the conceptual landscape of the smart museum field. The first presents a correlation matrix illustrating associations between individual publications and the identified attributes of the smart museum, encompassing both works focused on specific technological domains (e.g., IoT, XR, AI) and studies derived from the broader theoretical and institutional corpus addressing general aspects of contemporary or smart museums ( Figure 6). The second heatmap visualizes the temporal distribution of the same publications, tracing the evolution of conceptualizations related to smart museum attributes over time ( Figure 7). Together, these visualizations map thematic and temporal co-occurrences, revealing prevailing conceptual emphases within the literature. Although based on an extensive rather than systematic review, the identified patterns reflect the exploratory and qualitative nature of the analysis, providing an interpretive perspective on the material examined.

At the synthesis level, a conceptual model of the smart museum was developed, defining its principal categories and characteristics that approximate its ideal form. The findings were visualized using two diagrams ( Figures 1, 8)—a definition diagram and a conceptual diagram of the smart museum concept—and further elaborated through three tables, which present, categorize, and detail the core attributes shaping its profile ( Tables 1- 3). In total, fifteen attributes were identified and synthesized into a conceptual framework portraying the smart museum through a cultural lens, providing a foundation for guiding future smart museum practices.

## Findings

**Figure 2.**
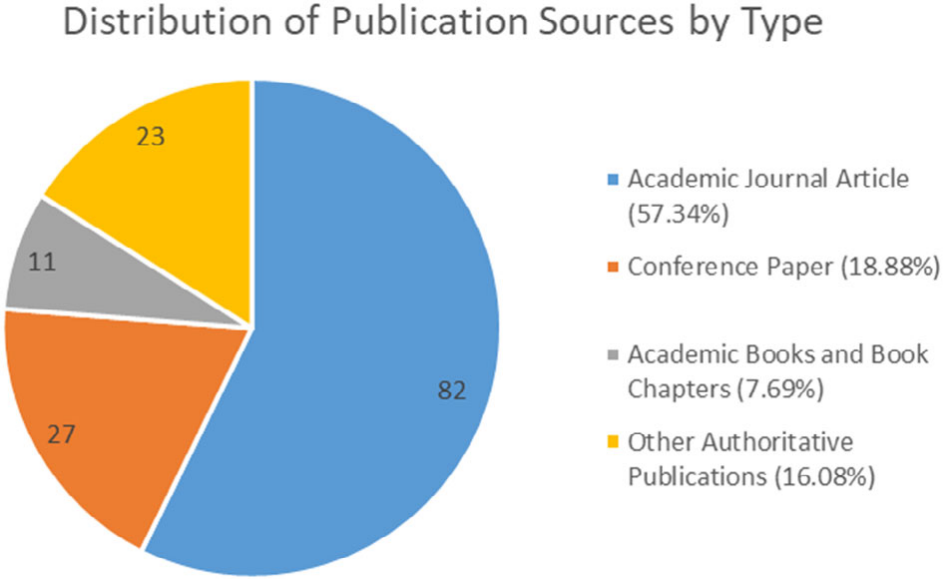
Types of publications studied.

The analysis of the collected material first enabled the identification of the typological differentiation of the examined publications. A detailed pie chart ( Figure 2) illustrates the proportional distribution of sources by publication type, encompassing academic journal articles, conference papers, academic books and book chapters, and other authoritative publications. The distribution reveals a clear methodological preference for primary, peer-reviewed research outputs. This tendency is particularly evident in the predominance of academic journal articles (82 occurrences), which, together with conference papers (27) and academic books (11), collectively account for 83.92% of all sources examined.

**Figure 3.**
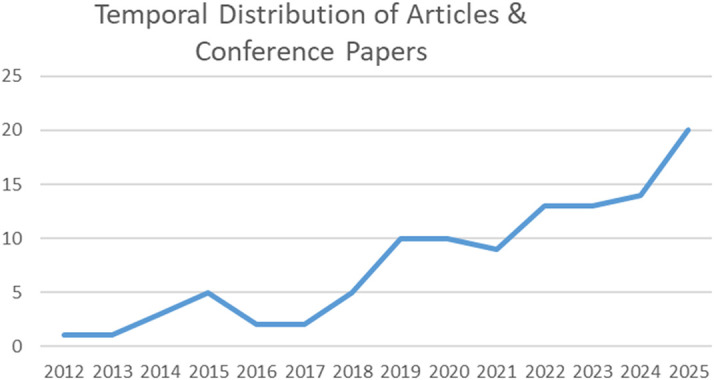
Temporal distribution of peer-reviewed articles.

**Figure 4.**
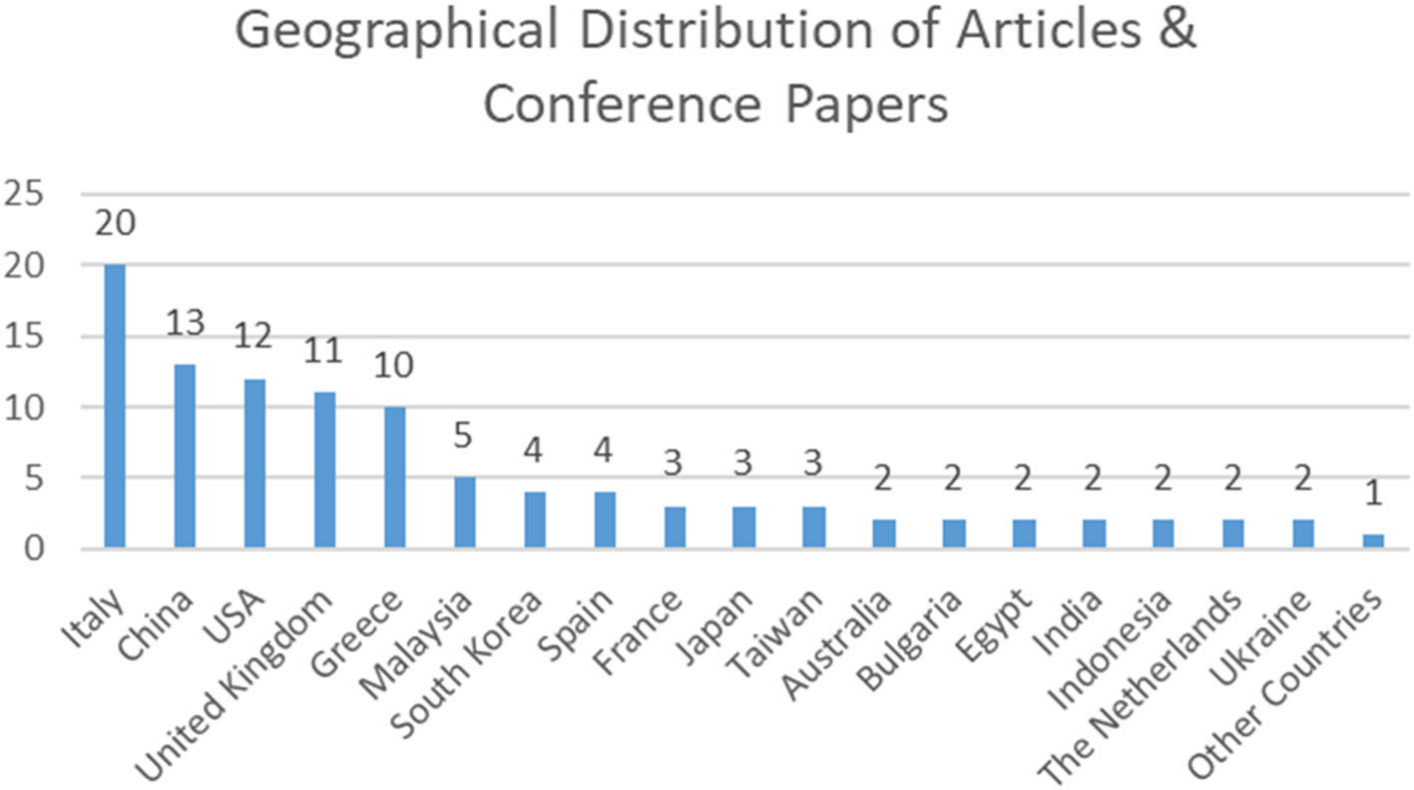
Geographical distribution of peer-reviewed articles.

Subsequently, the temporal and geographical distribution of academic journal articles and conference papers was analyzed ( Figures 3– 4). As shown in Figure 3, scholarly interest in the smart museum has exhibited a marked upward trend since 2017–2018. Between 2022 and 2025 alone, 60 publications were produced—representing 60% of the total corpus—indicating a significant surge in research activity. This progression demonstrates that the study of smart museums has evolved into a sustained and expanding field of inquiry rather than a sporadic or fragmented research phenomenon.

Geographically, the distribution of publications reveals a notable concentration in specific regions. Italy (20 publications), China (13), and the United States (12) lead in output, followed by the United Kingdom (11) and Greece (10). Countries with moderate levels of production contributed between three and five publications, while lower-volume contributors accounted for two each ( Figure 4). A diverse “long tail” of twenty countries—including Austria, Bosnia and Herzegovina, Canada, Croatia, Cyprus, Iran, Israel, Kazakhstan, Lithuania, Norway, Pakistan, Poland, Portugal, Saudi Arabia, Scotland, Singapore, Slovakia, Switzerland, Turkey, and Vietnam—each produced a single publication. This pattern indicates a globally distributed yet uneven research landscape, characterized by a few dominant hubs of production and numerous emerging contributors.

**Figure 5.**
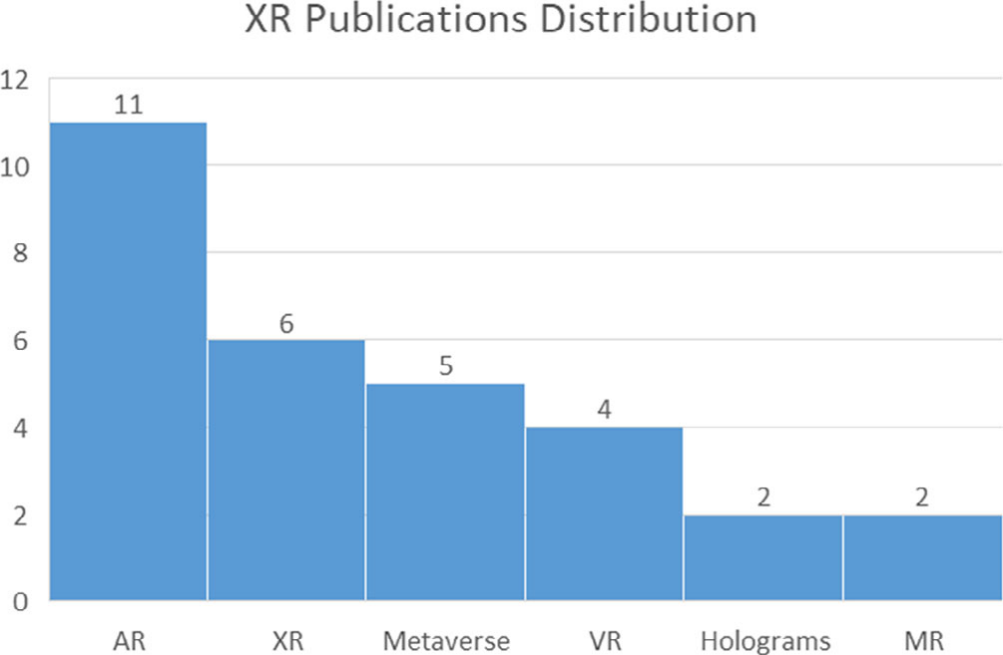
Publications across the XR Spectrum.

The literature review further identified seven principal technology categories. Among these, 33 research outputs broadly addressed digital technologies, complemented by more specific focuses on XR (30), Fusion Technologies (22), AI (18), IoT (8), Robotics (6), and Blockchain (1). A small subset concerned analog or non-digital technologies (3), while 22 additional publications and institutional sources constituted the theoretical-institutional corpus. Within the 30 XR-related studies, research predominantly focused on Augmented Reality (AR) applications (11), underscoring its growing importance in enhancing museum engagement, interaction, and interpretation ( Figure 5).

**Figure 6.**
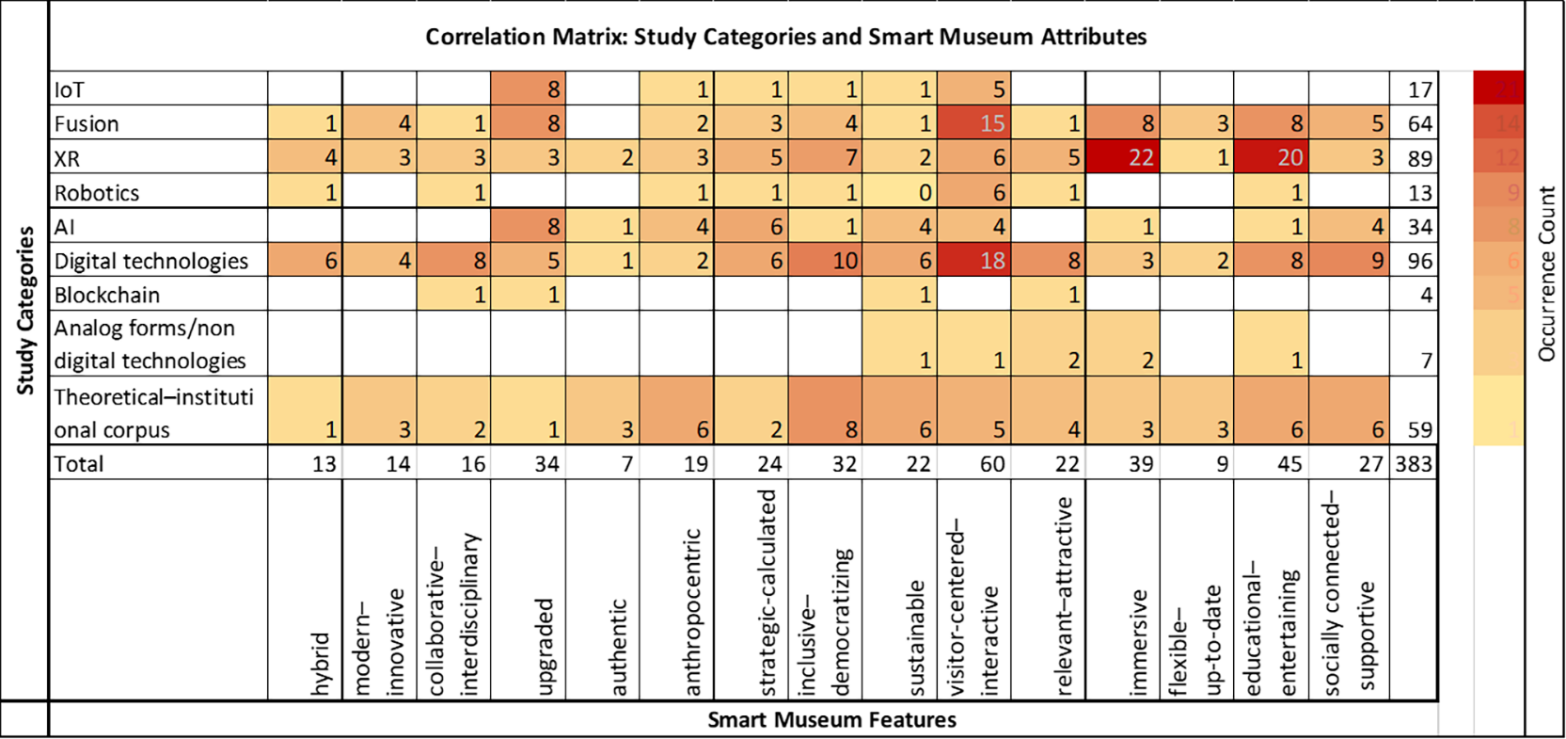
References to smart museum attributes by publication type.

Based on the established study categories, the analysis correlated the frequency of research references with fifteen proposed smart museum attributes ( Figure 6), enabling the identification of patterns and interconnections between them. Although the overall review demonstrated that these attributes are deeply interdependent and mutually reinforcing, for analytical clarity and interpretive depth, each attribute was examined individually to facilitate a critical understanding of its nature, function, and contribution to the overarching concept of the smart museum.

The Correlation Matrix ( Figure 6) revealed several noteworthy associations. Technologies under the XR spectrum show strong correlations with the attributes of immersiveness (22 references) and educational–entertaining character (20), underscoring XR’s role in enriching visitor engagement and learning. Digital technologies display the highest association with the visitor-centered–interactive attribute (18), and moderate to high correlations with inclusivity–democratization (10) and social connectedness–relational support (9). Artificial Intelligence (AI) exhibits its strongest linkage with the upgraded attribute (8), reflecting its transformative role in optimizing museum operations and visitor interaction.

More broadly, the sources collectively associate the smart museum most prominently with visitor service enhancement (60 references), followed by the educational and entertaining role of the museum (45). High frequencies were also recorded for immersiveness (39), overall functional upgrading (34), inclusivity and democratization of cultural content (32), and social connectedness (27). Other frequently cited attributes include strategic management (24), sustainability (22), attractiveness (22), human-centered orientation (19), and interdisciplinary collaboration (16). These findings depict the smart museum as an institution increasingly defined by its dynamic engagement with visitors, its educational and participatory mission, and its responsiveness to societal needs.

**Figure 7.**
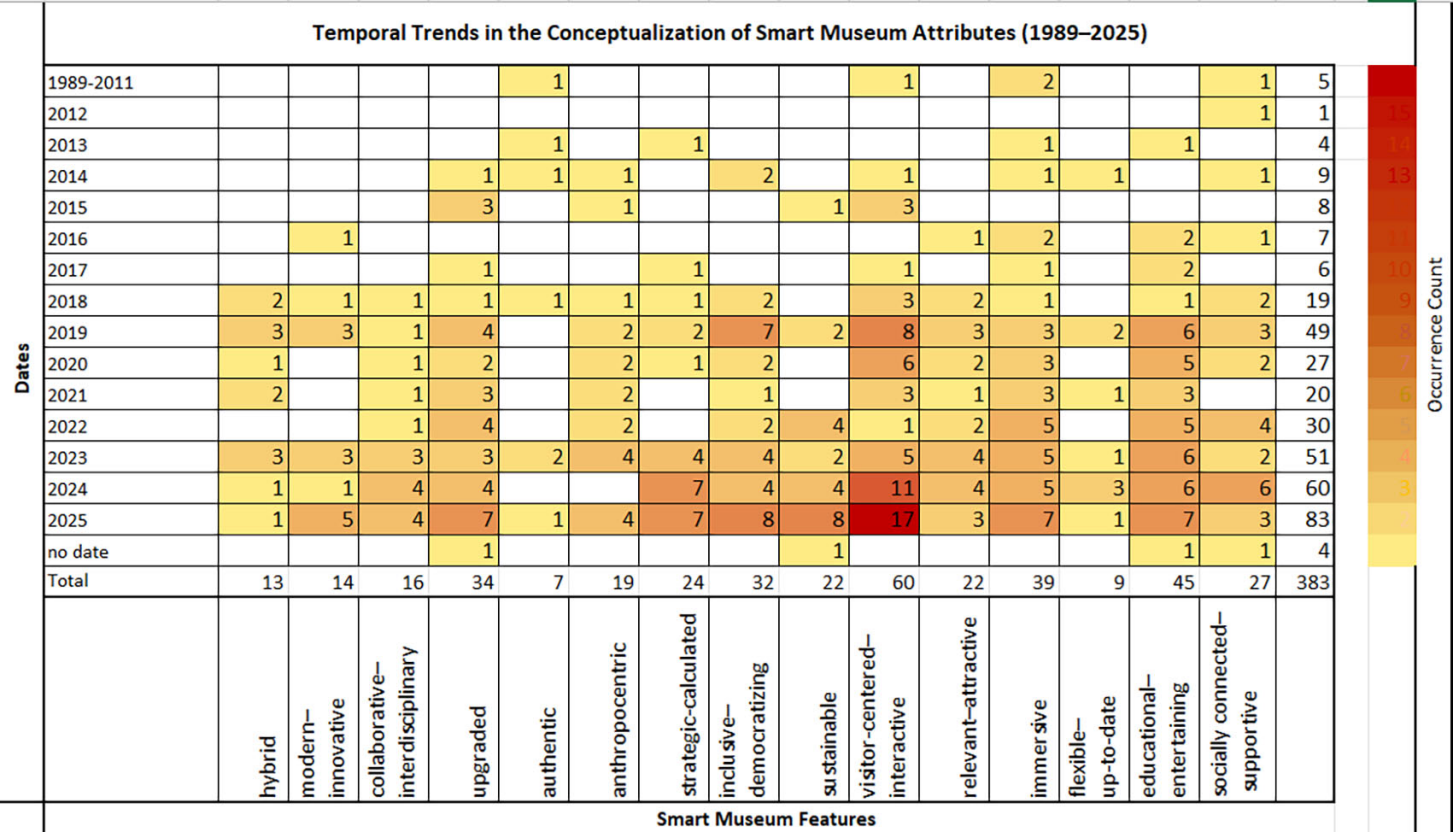
Temporal trends in smart museum attributes.

A complementary correlation matrix ( Figure 7) visualizes the temporal distribution of references to the identified smart museum attributes, quantifying their evolution from 1989 to 2025. Beyond the evident intensification of references after 2018—consistent with the trends observed in Figure 3—the matrix traces the introduction of “sustainability” around 2015 and its maturation between 2022 and 2025. It also highlights the stability of visitor-centered, educational, and immersive attributes across the examined period, as well as the relatively limited yet conceptually significant and consistent emphasis on the museum’s social connectedness with its wider community.

The findings, visualised in Table 1, provide a consolidated representation of what the smart museum of the future might be. They illuminate how the digital transformation of the museum reshapes its structure, rendering it hybrid, innovative, and technologically upgraded, while ephasizing the necessity of collaboration and interdisciplinarity to keep up with the digital age.

They also demonstrate the positive impact of technology on the visitor experience, as it provides personalization, interactivity, and participation opportunities, and its power to create immersive and engaging worlds that transform the traditional museum into a new, relevant, and engaging one for visitors, especially the youth, provided it stays in touch with their needs and ensures an unobstructed and discreet technological mediation.

The study also indicates that the integration of new and emerging technologies leads to a new type of educational-entertainment museum character, capable of facilitating visitors’ critical reflection and personal understanding of cultural heritage (CH) and at the same time socializing their experience and contributing to knowledge sharing and public awareness. Finally, they identify a challenge that technology-mediated experiences in museums can pose, namely the risk of altering or losing the sense of authenticity and neglecting the museum’s objectives, underscoring the need for smart museums to adopt a strategic-calculated approach and ensure both their sustainability and their catalytic role in the democratization of culture.

**Table 1.** The Smart Museum Attributes Based on Literature Findings.

Attributes	Description	Author/s
hybrid	Museums no longer operate solely in the physical environment but are also expanding into the digital space, functioning either as platforms for digital exhibitions and collections or as environments for visitor dialogue and engagement tools. The visiting experience thus becomes holistic - before, during and after the physical visit - and ubiquitous, fostering global visibility.	Black 2018, Ming 2018, Bowen and Giannini 2019, Khadraoui 2019, Sarakinos & Lembessis 2019, Margetis et al. 2020, Nisiotis and Alboul 2021, Simone, Cerquetti, and La Sala 2021, Hutson and Hutson 2023, Calvi & Vermeeren 2023, Modliński, Fortuna, and Rożnowski 2023, Philippopoulos et al. 2024, Ahmed, Azmy & Zaki 2025.
modern-contemporary innovative	Smart museums conceptualise themselves as “contemporary institutions” by embracing digital experimentation and innovation to bridge the past and the future, the tangible and the intangible. This progressive approach enables dynamic and engaging interpretations of history and art, fostering creative narratives enriched with interactive features that appeal to diverse audiences across genders, ages, and cultures. Their contemporary identity is often embodied in the redesign of public spaces, a key step in redefining these museums as hybrid entities.	Cerquetti 2016, Manna & Palumbo 2018, Bowen and Giannini 2019, Recupero et al. 2019, Sarakinos & Lembessis 2019, Scott, Salili-James, and Smith 2023, Fernandes & Casteleiro-Pitrez 2023, Pérez 2023, Shi, Ghafar & Yahaya 2024, Ahmed, Azmy & Zaki 2025, Ismail, Mat Som και Hanafiah 2025, Kung & Lin 2024, Yang & Huang 2025.
collaborative/interdisciplinary	The technological requirements and opportunities of the post-pandemic era necessitate collaborations and the integration of new disciplines and specialties within the museum environment. Interdisciplinary cooperation—linking Museology, Technology, and Psychology, alongside curators, conservators, and IT specialists—forms the core mechanism for designing digital experiences and managing hybrid, user-centered operations. Equally crucial is the smart museum’s collaboration with its staff, fostering engagement and mission alignment, and with its public, enhancing participation, creativity, dialogue, and critical reflection.	Black 2018, Bowen & Giannini 2019, Ludden and Russick 2020, Zachila et al. 2021, Mucchi, Milanesi, and Becagli 2022, Fernandes and Casteleiro-Pitrez 2023, Longo & Faraci 2023, Modliński, Fortuna, and Rożnowski 2023, Avlonitou & Papadaki 2024b, Li et al. 2024, Philippopoulos et al. 2024, Wang 2024, Cipparrone et al. 2025, Ivanov & Velkova 2025, Kou & Chen 2025, Nguyen et al. 2025.
upgraded	Digital technologies enhance the supervision of management operations and processes, rendering them increasingly automated, interconnected, efficient, and faster. They optimize all institutional functions—from environmental performance and preventive conservation to research and curation—while elevating visitor engagement through interactive exhibits, dynamic navigation, and the digital reconstruction of artifacts and historical contexts.	Chianese and Piccialli 2014, Brondi and Carrozzino 2015, Chianese, Piccialli, and Valente 2015, Mighali et al. 2015, Ciecko 2017, Dossis et al. 2018, Bhattacharya 2019, Sarakinos & Lembessis, 2019, Siountri, Skondras, and Vergados 2019, Summers 2019, Spachos and Plataniotis 2020, Varitimiadis et al. 2020, Khan et al. 2021, Simone, Cerquetti, and La Sala 2021, Wang 2021, Bobasheva, Gandon, and Precioso 2022, Frank and Frank 2022, Gaugne et al. 2022, Mucchi, Milanesi, and Becagli 2022, Cai, Zhang, and Younghwan 2023, Liu and Guo 2023, Lu et al. 2023, Philippopoulos et al. 2024, Shi, Ghafar & Yahaya 2024, Tresnawati et al. 2024, Wang 2024, Avlonitou, Papadaki & Apostolakis 2025, Daineko et al. 2025, Islam et al. 2025, Ozdemir & Zonah 2025, Puspasari, Siradjuddin & Rachmansyah 2025, Xu & Zhou 2025, Yu et al. 2025, “How Wireless Data Loggers,” n.d.
visitor-centered–interactive	The smart museum, attuned to the diversity of its visitors’ needs, interests, expectations, and available time, provides a meaningful experience through its interactive, participatory, and even co-creative nature, supported by tailored guidance and recommendations. This approach fosters a participatory culture that entails new power dynamics, encouraging visitors to engage with, generate, and influence cultural content, thereby enabling pluralistic interpretations of heritage. The visitor-centered model maximizes engagement, satisfaction, and deeper understanding, supporting personalized, self-directed learning and entertainment. In the physical domain, creating a supportive, hospitable, and comfortable environment addresses visitors’ practical and psychological needs, directly promoting visits and elevating the overall dynamic experience.	Chen, Ho & Ho 2006, Leue, Jung, and tom Dieck 2014, Chianese, Piccialli, and Valente 2015, Lupetti, Germak, and Giuliano 2015, Mighali et al. 2015, Palombini 2017, Black 2018, Dossis et al. 2018, Kyprianidou and Papadaki 2018, Bowen and Giannini 2019, Ch’ng et al. 2019, Iio et al. 2019, Frost, Thomas, and Forbes 2019, Khadraoui 2019, Papadaki 2019, Recupero et al. 2019, Saggese, Vento, and Vigilante 2019, Bessaa, Levillain, and Tijus 2020, Kirova 2020, London 2020, López-Martínez, Carrera, and Iglesias 2020, Margetis et al. 2020, Spachos and Plataniotis 2020, Fuentes-Moraleda et al. 2021, Khan et al. 2021, Wang 2021, ICOM 2022, Hutson and Hutson 2023, Kayukawa et al. 2023, Komarac and Došen 2023, Liu and Guo 2023, Lu et al. 2023, Avlonitou and Papadaki 2024a, Ivanov 2024, Li et al. 2024, Mao, Cao & Yu 2025, Ismail, Nessim & Fathy 2024, Philippopoulos et al. 2024, Shi, Ghafar & Yahaya 2024, Tresnawati et al. 2024, Wang 2024, Xu & Pan 2024, YiFei and Othman 2024, Ahmed, Azmy & Zaki 2025, Avlonitou, Papadaki & Apostolakis 2025, Daineko et al. 2025, Derda & Predescu 2025, Cipparrone et al. 2025, Islam et al. 2025, Ismail, Mat Som και Hanafiah 2025, Ivanov & Velkova 2025, Kim & Kim 2025, Kung & Lin 2024, Nguyen et al. 2025, Ozdemir & Zonah 2025, Puspasari, Siradjuddin & Rachmansyah 2025, Shlyakhetko et al. 2025, Xu & Zhou 2025, Yang & Huang 2025, Yu et al. 2025.
relevant-attractive	Aligned with its visitor-oriented mission, the smart museum prioritizes personal relevance and belonging through active participation and emotional engagement. Storytelling and gamification transform fragmented knowledge into coherent meaningful narratives. Understanding visitor segments enables experiences that are intellectually stimulating, emotionally fulfilling, and personally connected to CH. Digital technologies underpin this strategy, attracting and engaging audiences —particularly younger demographics—and fostering loyalty.	Cerquetti 2016, Black 2018, Manna & Palumbo, 2018, Bowen & Giannini 2019, Recupero et al. 2019, Hughes and Moscardo 2019, Cagliostro 2020, London 2020, Zollo et al. 2021, Mucchi, Milanesi, and Becagli 2022, Zhang and Abd Rahman 2022, Fernandes and Casteleiro-Pitrez 2023, Hutson and Hutson 2023, Longo and Faraci 2023, Lu et al. 2023, Avlonitou & Papadaki 2024b, Li et al. 2024, Philippopoulos et al. 2024, Xu & Pan 2024, Ahmed, Azmy & Zaki 2025, Ismail, Mat Som και Hanafiah 2025, Yang & Huang 2025.
provider of immersive experiences	Delivering immersive experiences is a core attribute of the modern smart museum, motivated by the pursuit of aesthetic engagement and visitors’ desire for escapism. This is achieved through high-quality content mediated by intuitive, seamless, and non-intrusive technologies—primarily XR and gamified interfaces—that foster emotional and cognitive engagement. Drawing on Csikszentmihalyi’s flow theory, the museum aspires to elicit a state of deep absorption and holistic satisfaction. This engagement—manifested through emotion, curiosity, and a strong sense of presence—anchors knowledge in memory. Such experiences move beyond passive observation, transporting visitors across time and space and promoting both intellectual insight and emotional resonance.	Davis 1989, Csikszentmihalyi 1990, Pine and Gilmore 2013, Leue, Jung, and tom Dieck 2014, ANTEPRIMA La Città Proibita VR” 2016, Mason 2016, Marques 2017, Ming 2018, Harrington 2019, Katz 2019, Recupero et al. 2019, Cagliostro 2020, London 2020, Margetis et al. 2020, Nisiotis and Alboul 2021, Trunfio et al. 2021, Wang 2021, Gaugne et al. 2022, Rizvic et al. 2022, Trunfio et al. 2022, Zhang and Abd Rahman 2022, Fernandes & Casteleiro-Pitrez 2023, Gao and Braud 2023, Hutson and Hutson 2023, Komarac and Došen 2023, Longo & Faraci, 2023, Yang 2023, Avlonitou & Papadaki 2024b, Li et al. 2024, Mao, Cao & Yu 2025, Shi, Ghafar & Yahaya 2024, YiFei and Othman 2024, Jangra et al. 2025, Kung & Lin 2024, Nguyen et al. 2025, Shlyakhetko et al. 2025, Xu & Zhou 2025, Yang & Huang 2025, Yu et al. 2025.
flexible/up-to-date	Flexibility defines the smart museum, fostered by ongoing content updates, technological enhancements, and up-to-date additions that sustain its relevance and help avoid isolation. By engaging continuously with its community and adopting a modular, adaptable structure, it can diversify cultural offerings and efficiently redeploy resources. Societal awareness and technological agility ensure it remains accessible, responsive, and aligned with evolving contexts.	Desvallées & Mairesse 2014, Bowen & Giannini 2019, Khadraoui 2019, Zachila et al. 2021, Longo & Faraci 2023, Philippopoulos et al. 2024, Shi, Ghafar & Yahaya 2024, Xu & Pan 2024, Kou & Chen 2025.
educational – entertaining	Smart museums combine education and recreation (edutainment) to promote self-directed exploration, using interactive, hands-on, and sensory experiences alongside gamification and storytelling to link emotion with knowledge. This balance prevents excessive entertainment or information overload while stimulating curiosity and critical thinking. Leveraging AR, AI, and other digital tools, they enhance memory retention and the hedonic value of visits. By fostering co-creation and informal learning in inclusive, non-hierarchical environments, smart museums make cultural knowledge accessible, engaging, and personally meaningful, enabling visitors to actively participate in interpreting content while respecting curatorial guidance and pluralistic collective memory.	Pine and Gilmore 2013, “ANTEPRIMA La Città Proibita VR” 2016, Cerquetti 2016, Marques 2017, Palombini 2017, Black 2018, Bowen & Giannini 2019, Harrington 2019, Khadraoui 2019, Katz 2019, Sarakinos & Lembessis 2019, “Seeing Impressionism” 2019, Cagliostro 2020, Kasperiuniene and Tandzegolskiene 2020, López-Martínez, Carrera, and Iglesias 2020, Margetis et al. 2020, “MuseumsQuartier Wien” 2020, Liestøl 2021, Trunfio et al. 2021, Wang 2021, ICOM 2022, Lee, Park, and Lee 2022, Pan, Zhang, and Yang 2022, Rizvic et al. 2022, Trunfio, Jung & Campana, 2022, Calvi and Vermeeren 2023, Fernandes and Casteleiro-Pitrez 2023, Hutson and Hutson 2023, Longo & Faraci, 2023, Modliński, Fortuna, and Rożnowski 2023, Gao and Braud 2023, Avlonitou & Papadaki 2024b, Li et al. 2024, Mao, Cao & Yu 2025, Philippopoulos et al. 2024, Shi, Ghafar & Yahaya 2024, YiFei and Othman 2024, Daineko et al. 2025, Kim & Kim 2025, Kung & Lin 2024, Ozdemir & Zonah 2025, Puspasari, Siradjuddin & Rachmansyah 2025, Xu & Zhou 2025, Yu et al. 2025, “Interactive Holograms” n.d.
socially connected and relationally supportive	Smart museums counter the isolating effects of technology by using it to enhance the inherently social nature of museum experiences. They address people’s needs for networking, interaction, and collective engagement, fostering togetherness through collaborative participation, crowdsourcing, and community dialogue both on-site and online. By employing social media and AI-driven clustering, they cultivate lasting, trust-based relationships, build online and local communities, mobilize participation, and create shareable moments that generate positive digital engagement. This fosters intercultural dialogue and civic awareness, turning visitors from passive observers into active cultural participants.	Rentschler & Radbourne 2008, Fletcher & Lee 2012, Rahimi 2014, Mason 2016, Black 2018, Kyprianidou and Papadaki 2018, Bowen and Giannini 2019, OECD/ICOM 2019, Papadaki 2019, Hourdakis and Ieronimakis 2020, Margetis et al. 2020, Behera & Gangadhar 2022, ICOM 2022, Olaz et al. 2022, Refae 2022, Calvi and Vermeeren 2023, Longo & Faraci 2023, Avlonitou and Papadaki 2024a, Avlonitou & Papadaki 2024b, Boiano et al. 2024, Ivanov 2024, Li et al. 2024, Xu & Pan 2024, Derda & Predescu 2025, Ismail, Mat Som και Hanafiah 2025, Yu et al. 2025, “Culture in Crisis · V&A” n.d.
authentic	Possessing equally valid and authentic cultural content, the smart museum must leverage its comparative advantages and tailor a distinctive profile, character, and identity based on its needs. This strategic differentiation prevents imitation, allowing the museum to offer authentic, non-standardized experiences. Since originality and authenticity increasingly drive visitor expectations in the “experience economy,” and as extended technologies redefine what is “real,” safeguarding the authenticity and intrinsic value of exhibits becomes a critical priority.	Rentschler and Radbourne 2008, Pine and Gilmore 2013, Desvallées & Mairesse 2014, Ming 2018, Calvi and Vermeeren 2023, Longo & Faraci, 2023, Derda & Predescu 2025.
anthropocentric	The smart museum adopts a strictly anthropocentric approach, treating technology as a means to serve people and advance social development rather than an end in itself. It avoids overemphasis on virtual realities, which can diminish value, erode the artistic aura of heritage, and divert attention from the museum’s core mission. Guided by the principles of Digital Humanism, it upholds human autonomy, safeguards democratic values against algocracy, and channels human intelligence into cultural creation. Ultimately, its design remains people-centered, fostering meaningful experiences that enhance well-being, create genuine value and prioritize ethical responsibility over mere technological progress.	Floridi 2014, De Angeli and O'Neill 2015, Ming 2018, Bowen and Giannini 2019, Tassis 2019, Bessaa, Levillain, and Tijus 2020, Kirova 2020, Wang 2021, Zachila et al. 2021, Chu et al. 2022, ICOM 2022, Fernandes and Casteleiro-Pitrez 2023, Modliński et al. 2023, Bunz 2023, Thiel 2023, Avlonitou, Papadaki & Apostolakis 2025, Cipparrone et al. 2025, Derda & Predescu 2025, Puspasari, Siradjuddin & Rachmansyah 2025.
adopter of strategic-calculated approach	The smart museum adopts a strategic, calculated approach, aligning innovation with human values, ethical principles, institutional mission, and sustainability. It utilizes a holistic framework, acknowledging the interdependence of all museum functions so that improvements in one area strengthen others. This approach involves integrating data-driven insights—from visitor behavior analysis to facility optimization and artifact preservation—with continuous evaluation and adaptive management. It also employs relational marketing to convert data into meaningful relationships, thus enhancing planning, communication, and overall institutional effectiveness.	Neuhofer, Buhalis, and Ladkin 2013, Ciecko 2017, Huang and Rust 2018, CAIML, 2019; Recupero et al. 2019, Ludden and Russick 2020, Hutson & Hutson 2023, Longo & Faraci 2023, Modliński, Fortuna, and Rożnowski 2023, Thiel 2023, Avlonitou and Papadaki 2024a, Ivanov 2024, Li et al. 2024, Philippopoulos et al. 2024, Slyusar, Nikitenko, & Voronkova 2024, Xu & Pan 2024, YiFei and Othman 2024, Avlonitou, Papadaki & Apostolakis 2025, Derda & Predescu 2025, EUDHIT, 2025, Islam et al. 2025, Kou & Chen 2025, Ozdemir & Zonah 2025, Puspasari, Siradjuddin & Rachmansyah 2025.
Inclusive & democratizing	The smart museum is inclusive and democratizing, promoting cultural participation through diversity, equity, and broad accessibility, for both its staff and audience. It employs digital tools and inclusive design, it provides comfortable, sensory-friendly and multilingual interactive experiences that make cultural heritage understandable and engaging for all, including marginalized groups and those with disabilities. In an era of challenged democratic values, the museum actively upholds diversity, inclusion, and justice, while enhancing user trust through ethical design and respect for data privacy, thereby transforming access to culture into a shared democratic right.	Chianese and Piccialli 2014, Desvallées & Mairesse 2014, Black 2018, Dossis et al. 2018, Bowen & Giannini 2019, Harrington 2019, Hughes and Moscardo 2019, Khadraoui 2019, OECD/ICOM 2019, Recupero et al. 2019, Sarakinos & Lembessis 2019, Hourdakis and Ieronimakis 2020, Margetis et al. 2020, Simone, Cerquetti, and La Sala 2021, “V&A Annual Report 2020 to 2021” 2022, ICOM 2022, Hutson and Hutson 2023, “V&A Annual Report 2022 to 2023” 2023, Kayukawa et al. 2023, Fernandes and Casteleiro-Pitrez 2023, Avlonitou & Papadaki 2024b, Ivanov 2024, Li et al. 2024, Wang 2024, Ahmed, Azmy & Zaki 2025, Avlonitou, Papadaki & Apostolakis 2025, Cipparrone et al. 2025, Daineko et al. 2025, Nguyen et al. 2025, Ozdemir & Zonah 2025, Sterling 2024, Yu et al. 2025.
sustainable	The smart museum embraces sustainability by aligning with international standards that advance social, economic, and environmental development. Through digital innovation and synergistic technologies, it ensures the long-term preservation and accessibility of collections while minimizing its environmental footprint via optimized energy use, climate control, and reduced material reliance. Technology also facilitates its financial viability by enabling alternative funding models like e-commerce and crowdsourcing. Ultimately, the smart museum cultivates a mindset of ethical awareness, actively sensitizing audiences to environmental and social issues, including global inequalities, and fostering a culture of responsibility that serves present and future generations alike.	United Nations 2015, CAIML, 2019; OECD/ICOM 2019, “V&A Annual Report 2021 to 2022” 2022, Zhang et al. 2022, Mucchi, Milanesi, and Becagli 2022, ICOM 2022, Bunz 2023, Lu et al. 2023, Avlonitou and Papadaki 2024a, Ismail, Nessim & Fathy 2024, Li et al. 2024, Slyusar, Nikitenko, & Voronkova 2024, Ahmed, Azmy & Zaki 2025, Avlonitou, Papadaki & Apostolakis 2025, Bi, Song and Zhang 2025, EUDHIT, 2025, Islam et al. 2025, Kou & Chen 2025, Ozdemir & Zonah 2025, Puspasari, Siradjuddin & Rachmansyah 2025, “V&A: Sustainability”, n.d.

**Figure 8.**
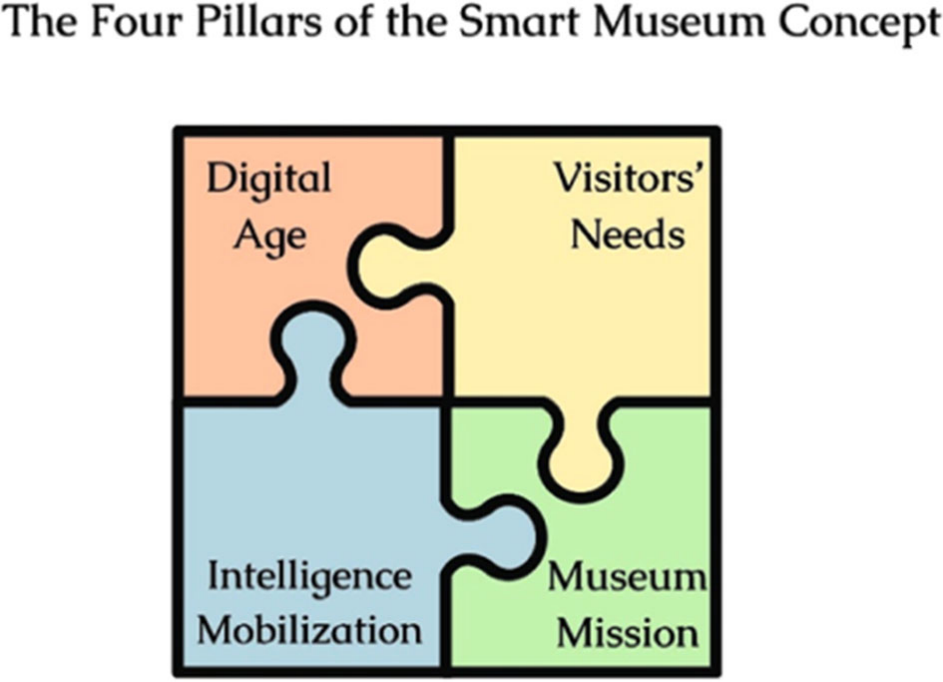
Smart museum conceptual diagram.

Ultimately, the findings demonstrate that the attributes of the smart museum are not discrete but interconnected, co-dependent, and mutually reinforcing. They interact dynamically within the conceptual pillars defined earlier (technological, visitor-centered, mission-driven, socio-cultural), forming a coherent, integrative framework—a “puzzle-like” structure in which each element contributes critically to shaping the comprehensive and integrative vision of the future smart museum, viewed through a cultural lens ( Figure 8).

## Discussion

### Looking to the future of the smart museum

Since the advent of the Internet in 1989, but mainly with the implementation of Web 2.0 and the spread of smartphones in the first decade of the 21st century, digital culture has accelerated rapidly and dominantly. In today’s post-digital age, where physical and digital lives are increasingly merged and interconnected, people are continuously sharing thoughts, images, and knowledge through the Internet, creating a global culture without physical boundaries. As digitization—initially of documents and now of our lives—becomes a central driver of human action, it transforms the way we exist, think, and communicate ( Black 2018, Bowen and Giannini 2019).

As digital culture permeates all social sectors, ICT tools are gradually reshaping museum management, practices, communication strategies, and the way museum exhibitions are designed. By integrating digital technologies at an increasing rate, museums are undergoing a digital transformation, where artificial agents, living organisms and human beings are redefined as informational entities within a unified, information-based space or ‘infosphere’ ( Floridi 2014). This infosphere, as Simone, Cerquetti, and La Sala (2021) argue, “reontologises” (i.e., intrinsically redefines) our world and the institutions within it, including museums.

Technology has thus become a vital enabler of museum development ( Khadraoui 2019). In the post-pandemic era, museums must adopt a digital mindset and strategic orientation to remain resilient and relevant ( Ludden & Russick 2020). Incorporating a digital dimension into exhibitions strongly predicts visitor engagement, particularly among technologically literate audiences who are more likely to support museums through financial means, electronic word-of-mouth (e-WOM), and online communities ( Zollo et al. 2021).

The integration of digital technologies enhances museums’ appeal, value creation, and experiential depth ( Cerquetti 2016, Recupero et al. 2019). Their interactive nature significantly increases attractiveness, particularly among younger audiences—Millennials and post-Millennials (Generations Y and Z)—for whom digital interactivity is a decisive factor in choosing to visit ( Manna & Palumbo 2018, Fernandes & Casteleiro-Pitrez 2023, Ismail et al. 2025). Consequently, museums are evolving from “boring, didactic, and inaccessible” institutions focused primarily on the past into engaging, participatory, and socially connected cultural spaces ( Hughes & Moscardo 2019, Longo & Faraci 2023).

This transformation simultaneously reinforces the educational mission of the smart museum. Learning now occurs in increasingly informal, multimodal contexts where visitors engage directly with exhibits in rich, multimedia, and multisensory environments ( Modliński et al. 2023). The convergence of technology and experiential education thus redefines museum pedagogy in the digital age.

A central element of this convergence lies in narrative and gamification. Narrative bridges creativity and scholarship, with digital storytelling enriching interactivity, immersion, and memory retention, especially among non-expert audiences ( Palombini 2017). When coupled with gamification, it promotes self-directed, choice-based learning and strengthens emotional engagement through feedback mechanisms ( Black 2018, Calvi & Vermeeren 2023). While analog forms of storytelling and gamification can also generate immersive learning ( Cagliostro 2020, London 2020), emerging technologies—which enhance self-directed learning ( Pan, Zhang & Yang 2022)—significantly strengthen presence, engagement, and satisfaction, fostering deeper and more personalized learning ( Yang & Huang 2025).

Immersive technologies (XR), especially when combined with AI (AIMR), transport visitors across times and spaces with remarkable fidelity, enriching the hedonic and emotional dimensions of the museum experience ( Yu et al. 2025). In this hybrid context, learning converges with enjoyment and entertainment—often valued even more than education ( Khadraoui 2019)—while their synthesis into edutainment adds further experiential and cultural value ( Trunfio et al. 2022).

However, technology devoid of conceptual grounding, purposeful design, and ethical awareness remains superficial and meaningless. In such cases, digital tools may lead to value co-destruction rather than co-creation, particularly when form supersedes content or when information is distorted ( Kirova 2020). Excessive technological mediation can also diminish the aesthetic and emotional impact of CH, undermining the authenticity of the museum experience ( Ming 2018).

The human factor, and particularly the role of the cultural manager, therefore remains indispensable. The implementation of technologies—especially AI—requires careful ethical governance to prevent bias, ensure minority representation, and promote responsible decision-making ( Bunz 2023, Thiel 2023, Boiano 2024, Avlonitou, Papadaki & Apostolakis 2025).

If two of the smart museum’s defining pillars—its entanglement with the digital age and responsiveness to audience needs—anchor it in the present, the other two—its humanistic orientation and sociocultural mission—ensure continuity. While visitor agency and participation define the new museum paradigm, maintaining a delicate equilibrium between participatory engagement and curatorial integrity remains crucial ( Derda & Predescu 2025).

In a global context marked by polycrisis, democratic erosion, and authoritarian resurgence, museums must reaffirm their commitment to diversity, inclusion, participation, sustainability, and justice to counter the decline of democratic values ( Sterling 2024). The hybrid and technologically advanced nature of the smart museum—through innovations such as personalization and multilingual support—enhances accessibility and inclusivity, extending participation to previously marginalized audiences ( Ahmed, Azmy & Zaki 2025). Notably, inclusivity now extends not only to visitors but also to museum staff, reflecting greater diversity, equity, and representation ( V&A 2022 pp. 6, 33).

Aligning the museum’s digital transformation with its human-centered mission requires a strategic, holistic, and data-driven approach, marking a shift from “intuitive” to “calculated” strategies ( Modliński et al. 2023).

Digital infrastructures such as IoT support predictive models that optimize artifact care, exhibition design, environmental management, and resource allocation ( Islam et al. 2025, Puspasari, Siradjuddin & Rachmansyah 2025). When integrated with AI, these systems enable advanced data collection and analysis, providing real-time tracking of visitor interest, behavior, and ratings, which allows museum administrators to make informed decisions on exhibit allocation and strategic planning ( Li et al. 2024, Philippopoulos et al. 2024). Continuous assessment of digital tools for effectiveness, inclusivity, and environmental impact remains essential ( Ozdemir & Zonah 2025). A holistic, interconnected strategy ensures that innovations in one area reinforce progress in others, fostering adaptive, evidence-based development ( Xu & Pan 2024).

The fusion of digital technologies—particularly AI with IoT ( Puspasari, Siradjuddin & Rachmansyah 2025) and AI with XR ( Shlyakhetko et al. 2025)—emerges as a key enabler of sustainability, enhancing both environmental performance and visitor satisfaction ( Bi, Song & Zhang 2025). IoT-based systems also facilitate vital conservation functions such as monitoring temperature, humidity, and lighting to protect sensitive artifacts ( Li et al. 2024, Islam et al. 2025). Nevertheless, long-term success depends on establishing clear standards for digital resource sustainability ( Kou & Chen 2025) and comprehensive strategic planning ( Ozdemir & Zonah 2025).

Finally, the social dimension of the smart museum—its dual role as an educational and socially connected institution—remains central to its mission. In an era defined by technological acceleration, museums can act as ethical arenas for public reflection, facilitating dialogue and helping audiences critically evaluate the cultural, societal, and ethical implications of emerging technologies, particularly AI ( Thiel 2023).

Considering these tectonic changes and the preceding analysis, the study concludes by summarizing the 15 key attributes defining the smart museum, aligning them with its definition. These attributes encapsulate its unique relationship with the digital age, its institutional mission, its audiences, and the broader communities it serves ( Table 2).

**Table 2.** The Smart Museum Profile: Key Attributes.

aligned with the digital age	aligned with its mission	aligned with visitors' needs	mobilizing individual and collective intelligence
• hybrid • modern-innovative • collaborative-interdisciplinary • upgraded	• authentic • anthropocentric • adopter of strategic-calculated approach • inclusive-democratic • sustainable	• visitor-centered–interactive • relevant and attractive to its audience • provider of immersive experiences • flexible and up-to-date	• educational-entertaining • socially connected and relationally supportive

These attributes—representing the ideal qualities of a holistic smart museum, which emerged through the analysis, processing, and synthesis of our literature findings and were organised into four thematic sections in our description—are further analyzed and illustrated in a summary table ( Table 3). The summary table provides a comprehensive overview of the ideal profile of the smart museum, detailing its main characteristics, indicative methods of implementation and activation in the modern hybrid museum (including means and methods of achievement), and the anticipated benefits of these implementations.

**Table 3.** The Smart Museum Profile Through a Cultural Lens: Detailed Description.

Smart museum profile (Key Attributes)			
	Attributes	Means/methods of achievement	Benefits
1	**aligned with digital age** • hybrid • modern (contemporary)-innovative • collaborative- interdisciplinary • upgraded	• is digitally transformed • is ubiquitous (before, during, and after the visit) • bridges the past with the present through emerging technologies (e.g. XR) • all its functions and operations become more automated and interconnected (e.g. through AI, IoT and Blockchain technologies) • collaborates with IT professionals, cultural organizations, and communities	• expansion of physical boundaries • revitalization of visitor experiences • strengthening of museum competitiveness • attraction of younger visitors • participatory network development • acceleration and efficiency of management
2	**aligned with its mission** • authentic • anthropocentric • adopter of strategic-calculated approach • inclusive-democratic • sustainable	• applies control mechanisms to balance authenticity and technological mediation (e.g. via ethics committees, conducting audience surveys) • maintains a balance between technology (e.g. XR, robots) and strategic planning, regulates ethical issues and serves people, society, and museum goals • applies smart solutions (e.g. by monitoring indicators) to optimize its functions (e.g. reduce its ecological impact) and achieve its objectives • promotes increased accessibility and audience participation for socially excluded or marginalized groups, while fostering diversity and equity • makes its collections accessible to all and preserves them for future generations • uses digital platforms to attract financial and professional capitals or promote alternative financing	• safeguarding and implementation of museum mission • ensuring the leading role of humans in digital transformation • production of added value • contribution to democratization of culture and society • facilitation of museum's sustainability • contribution to environmental, social, and economic development
3	**aligned with visitors' needs** • visitor-centered–interactive • relevant and attractive to its audience • provider of immersive experience • flexible and up-to-date	• uses personalization and interactivity techniques to be accessible and comprehensible to all (e.g. via IoT, AI or robots/chatbots) • creates participatory and meaningful experiences/indelible memories (e.g. through “serious games” and XR technologies) • produces a sense of belonging to its audiences (e.g. through digital relationship marketing) • provides high-quality and renewable content via immersive technologies • ensures that the technology is seamless, minimally intrusive and easy to use • remains in touch with reality/visitors' needs (e.g. audience surveys via SM)	• enhancement of visitor experience • production of a state of “flow” and excitement • generation of overall visitor satisfaction • visitor's engagement with CH • integration of museums into everyday life • cultivation of a sense of belonging • support to the museum (e.g. through e-WOM) • retention of existing audiences
4	**mobilizing individual and collective intelligence** • educational-entertaining • socially connected and relationally supportive	• is experiential (e.g. provides immersive, and participatory experiences through XR, or activates engagement via SM) • uses storytelling and gamification techniques (on site or online) • encourages visitor-generated content (e.g. through SM) • allows tailored content (e.g. through personalized museum guides) • reevaluates hierarchies by abandoning the voice of authority and encouraging public's participation (e.g. via museum sites or SM) • offers individual time management (offers opportunities for “stagnant time”) • uses relationship marketing, SM, digital platforms or conferences to foster dialogue, sharing, social interaction and citizen involvement/participation	• reevaluation of the museum as a reference entity • awakening of curiosity • activation of informal and self-directed learning • stimulation of thought and reflection • promotion of empathy and co-creativity • development of long-term relationships of trust and mutual benefit • strengthening of bonds between museums and the local community • reinforcement of social cohesion • empowerment of collective intelligence for societal benefit

Thus, the smart museum is outlined as a hybrid, ubiquitous and modern entity that spans space and time, experimenting with and fully adopting smart solutions through both established (e.g., SM) and emerging technologies of the digital era (e.g., XR, IoT, AI, robots, holograms). It remains contemporary, innovative, and upgraded in all its operations—primarily automated, interconnected, and capable of delivering faster services and more efficient management. In this context, it forges collaborations with similar entities and technology consultants, while embracing new disciplines beyond traditional curatorship to acquire skills essential for meeting the modern needs of the post-pandemic era.

At the same time, the smart museum safeguards the authenticity and inherent values of CH from trivialization, offers genuine experiences to its visitors, and promotes digital humanism by balancing the virtual and the real. It preserves its mission and emphasises the human element in the museum experience. Consequently, it fosters the democratization of culture for all individuals while adopting a strategic-calculated approach and leveraging smart technological tools and solutions to ensure sustainability and contribute to sustainable development.

As a visitor-centered organization, the modern hybrid and participatory museum employs various techniques and technologies to meet the needs and desires of its visitors, continually reforming to stay viable and relevant. It strives to offer multisensory, immersive experiences and create lasting memories, focusing on the perceived usefulness and ease of use of technological tools while delivering satisfying and engaging CH content.

Simultaneously, the smart museum establishes a participatory culture within its context by encouraging visitor-generated content and co-production, particularly among younger audiences, treating them as “prosumers”—both consumers and producers ( Lee, Park, and Lee 2022). This approach stimulates curiosity, reflection, and empathy, thereby activating both cognitive and emotional skills.

 In the same vein, it involves audiences in informal and lifelong museum learning based on personal goals. It uses both analogical and digital means to facilitate the transmission of information, interpretation, and collective memory as an “open work” ( Cerquetti 2016) to boost social interaction and strengthen the bonds between museum and local communities, by activating their participation and knowledge.

## Conclusions

The rapid technological developments in computer science that have escalated over the past 20-25 years, along with the deliberate and growing adoption of smart technologies by museum policymakers worldwide, underscore the necessity of studying the smart museum. This paper employs an extensive literature review and key theoretical tools to explore the smart museum concept, addressing three core research questions: its origins, definition, and profile. The study details its attributes based on the application of emerging technologies in 21st-century museums.

Investigating the first research question revealed that the origins of the smart museum are rooted in the extension of socio-economic developments affecting this century-old institution in the digital age. Key factors include the emergence of New Museology and cultural shifts, reduced state support, the decline of mass culture as a model of passive consumption, the development and adoption of a constructivist, interactive learning model, the recognition of a culture-centric development model, and the digital revolution. These changes are reflected in the museum’s current definition and its evolving institutional role in post-war society.

Secondly, the study identified the variety and fragmentation in the use of the term “smart” within the museum field. Efforts were made to establish a clear and comprehensive definition through a cultural lens, demonstrating that smart technologies are valuable tools for achieving the humanitarian goals of the museum rather than ends in themselves. The smart museum is thus approached holistically, extending not only within the museum through its mission and visitor experience, but also beyond, as it is shaped by the digital age and influences the intelligence of individuals and communities interacting with it.

Finally, tracing the museum of the future, an attempt was made to identify its key characteristics as study parameters and design components of a holistically smart museum, shaped through the utilization of new and emerging technologies. Potential limitations, risks, and future prospects were also addressed.

The smart museum profile was depicted through 15 attributes, grouped into four general categories that correlate the museum with the digital age, its mission, visitors’ needs, and the activation of individual and collective intelligence.

In examining the attributes of the ideal smart museum of the future, the cultural lens remains the primary and defining perspective. This lens acts as a qualitative differentiator, positioning the smart museum not merely as an aggregation of technologically enhanced functions, but as an institution whose true value lies in interpreting, contextualizing, and evaluating its cultural impact and broader societal role.

Ultimately, the smart museum concept achieves completeness and coherence only when its four foundational pillars are fully integrated and balanced, aligning technological advancement and visitor needs with the museum’s human-centered and social mission, and with its evolving role in fostering awareness and mobilizing collective intelligence within the complex and demanding context of globalization. Future case studies that adopt these attributes as analytical parameters or design principles will be instrumental for assessing this framework’s validity and advancing the conceptual evolution of the smart museum.

### Ethics and consent

Ethical approval and consent were not required.

## Data Availability

No data are associated with this article.

## References

[ref1] Adorno TW : *Σύνοψη της Πολιτιστικής Βιομηχανίας [Résumé about Cultural Industry]. Translated by Lefteris Anagnostou.* Athens: Alexandria Publications;2000. (Original work published 1989).

[ref123] Ahmed EOM Azmy NO Zaki AA : New Digital Interpretation Tools for Enhancing the Visitor Experience at the Royal Jewelry Museum. 2025;9(2):271–300. 10.21608/mfth.2025.454444 Reference Source

[ref2] ANTEPRIMA La Città Proibita VR – OCULUS RIFT: MAO. 2016. Accessed July 23, 2024. Reference Source

[ref3] Arendt H : *“Κοινωνία και Κουλτούρα” [Society and Culture]. Η κουλτούρα των μέσων. Μαζική κοινωνία και πολιτιστική βιομηχανία [MediaCulture. Mass Society and the Culture Industry].* Livieratos K Fragoulis D , editors. Athens: Alexandria Publications;1994.

[ref4] Avlonitou C Papadaki E : The Role of Social Media Messages in Cultural Communication: The Case Study of an Instagram Reel. *Online Journal of Communication and Media Technologies.* 2024a;14(2):e202415–e202415. 10.30935/ojcmt/14291

[ref124] Avlonitou C Papadaki E : Using extended reality technologies in modern museums. *Arts & Communication.* 2024b;3(1):3428. 10.36922/ac.3428

[ref125] Avlonitou C Papadaki E Apostolakis A : A human–AI compass for sustainable art museums: Navigating opportunities and challenges in operations, collections management, and visitor engagement. *Heritage.* 2025;8(10):422. 10.3390/heritage8100422

[ref5] Beer D Burrows R : Consumption, Prosumption and Participatory Web Cultures. *Journal of Consumer Culture.* 2010;10(1):3–12. 10.1177/1469540509354009

[ref6] Behera B Gangadhar MR : Museum and Pandemics a Cautionary Tale from History: Impact, Innovations, Learning from Crises. *Anthropology and Ethnology Open Access Journal.* 2022;5(2):1–8. 10.23880/aeoaj-16000193

[ref7] Bessaa H Levillain F Tijus C : The Making of Museum Works as Smart Things. *Understanding Human Behaviour in Complex Systems: Proceedings of the Human Factors and Ergonomics Society Europe Chapter 2019 Annual Conference.* 2020.

[ref8] Bhattacharya I : Review of Smart Showcase: A Gift of Internet of Things (IoT) to Museum. 2019. 10.13140/RG.2.2.19697.43368

[ref126] Bi R Song C Zhang Y : Green Smart Museums Driven by AI and Digital Twin: Concepts, System Architecture, and Case Studies. *Smart Cities.* 2025;8(5):140. Reference Source

[ref9] Black G : Meeting the Audience Challenge in the ‘Age of Participation.’. *Museum Management and Curatorship.* 2018;33(4):302–319. 10.1080/09647775.2018.1469097

[ref10] Bobasheva A Gandon F Precioso F : Learning and Reasoning for Cultural Metadata Quality: Coupling Symbolic AI and Machine Learning over a Semantic Web Knowledge Graph to Support Museum Curators in Improving the Quality of Cultural Metadata and Information Retrieval. *Journal on Computing and Cultural Heritage.* 2022;15:1–23. 10.1145/3485844

[ref127] Boiano S Borda A Gaia G : Ethical AI and Museums: Challenges and new directions. *In Proceedings of the EVA London 2024 (EVA 2024).* London, UK;2024:8–12. 10.14236/ewic/EVA2024.4

[ref11] Bourdieu P Darbel A Schnapper D : *The Love of Art: European Art Museums and Their Public.* Cambridge, UK: Polity Press;1991. Reference Source

[ref12] Bowen JP Giannini T : The Digital Future for Museums. *Museums and Digital Culture.* 2019;551–577. 10.1007/978-3-319-97457-6_28

[ref13] Brondi R Carrozzino M : ARTworks: An Augmented Reality Interface as an Aid for Restoration Professionals. *The Lecture Notes in Computer Science.* 2015;384–398. 10.1007/978-3-319-22888-4_28

[ref128] Bunz M : The Role of Culture in the Intelligence of AI.In *AI in Museums. Reflections Perspectives and Applications.* Thiel S Bernhardt J , editors. Edition Museum 74. Transcript: Bielefeld, Germany;2023; pp.23–29. 10.14361/9783839467107

[ref14] Cagliostro M : Art of Escape, Magic, and Immersive Storytelling: The Museum as a Limitless Escape Game. MW2020: MuseWeb 2020. 2020. Reference Source

[ref15] Cai PY Zhang K Younghwan P : Application of AI Interactive Device Based on Database Management System in Multidimensional Design of Museum Exhibition Content. *Research Square (Research Square).* 2023 June. 10.21203/rs.3.rs-3074947/v1

[ref129] CAIML-Centre for AI and Machine Learning : The Vienna Manifesto on Digital Humanism. TU Wien. 2019. Accessed October 10, 2025. Reference Source

[ref16] Calvi L Vermeeren A : Digitally Enriched Museum Experiences – What Technology Can Do. *Museum Management and Curatorship.* 2023;1–22. 10.1080/09647775.2023.2235683

[ref17] Cerquetti M : More Is Better! Current Issues and Challenges for Museum Audience Development: A Literature Review. *Journal of Cultural Management Policy.* 2016;6(1):30–43. Reference Source

[ref23] Ch’ng E Cai S Leow F-T : Adoption and Use of Emerging Cultural Technologies in China’s Museums. *Journal of Cultural Heritage.* 2019;37(May):170–180. 10.1016/j.culher.2018.11.016

[ref18] Chabouri-Ioannidou A : Στρατηγική Διαχείρισης των Πολιτιστικών Ιδρυμάτων [Management Strategy of Cultural Institutions]. *Πολιτιστική Πολιτική και Διοίκηση: Πολιτιστική Διαχείριση [Cultural Policy and Management: Cultural Management].* Patras: Hellenic Open University;2003;25–60.

[ref19] Chen HC Ho CK Ho MC : New Communication Model Museums|PDF|Constructivism (Philosophy of Education)|Meme. *Scribd.* 2006. Accessed July 22, 2024. Reference Source

[ref20] Chianese A Piccialli F : Designing a Smart Museum: When Cultural Heritage Joins IoT. *IEEE Xplore.* 2014. September 1, 2014. 10.1109/NGMAST.2014.21

[ref21] Chianese A Piccialli F Valente I : Smart Environments and Cultural Heritage: A Novel Approach to Create Intelligent Cultural Spaces. *Journal of Location Based Services.* 2015;9(3):209–234. 10.1080/17489725.2015.1099752

[ref22] Chu J Xi L Zhang Q : Research on Ethical Issues of Artificial Intelligence in Education. *Lecture Notes in Educational Technology.* 2022;101–108. 10.1007/978-981-19-5967-7_12

[ref24] Ciecko B : Examining the Impact of Artificial Intelligence in Museums – MW17: Museums and the Web 2017. 2017. Accessed July 24, 2024. Reference Source

[ref130] Cipparrone A Elbasheer M Longo F : Integrating Industry 4.0/5.0 Technologies for Accessible and Engaging Museum Experiences. *Procedia Computer Science.* 2025;253:3227–3234. 10.1016/j.procs.2025.02.047

[ref25] Corona L : Museums and Communication: The Case of the Louvre Museum at the Covid-19 Age. *Humanities and Social Science Research.* 2021;4(1):p15. 10.30560/hssr.v4n1p15

[ref26] Crooke E : Communities, Change and the COVID-19 Crisis. *Museum and Society.* 2020;18(3):305–310. 10.29311/mas.v18i3.3533

[ref27] Csikszentmihalyi M : Flow: The Psychology of Optimal Experience. *ResearchGate.* Harper & Row;1990. January 1990. Reference Source

[ref28] Culture in Crisis · V&A: Victoria and Albert Museum. n.d.Accessed July 22, 2024. Reference Source

[ref131] Daineko Y Bolatov Z Tsoy D : Augmented Reality for Cultural Heritage: Bridging History and Technology in Museums. CEUR Workshop Proceedings. 2025;4014: Short2. Accessed October 10, 2025. Reference Source

[ref29] Davis F : Perceived Usefulness, Perceived Ease of Use, and User Acceptance of Information Technology. *MIS Q.* 1989;13(3):319–340. 10.2307/249008

[ref30] De Angeli D O’Neill E : A Smartphone Headset for Augmented-Reality Experiences in Museums|MW 2015: Museums and the Web 2015. 2015. Accessed July 22, 2024. Reference Source

[ref132] Derda I Predescu D : Towards humancentric AI in museums: Practitioners’ perspectives and technology acceptance of visitor-centered AI for value (co-) creation. *Museum Management and Curatorship.* 2025;40:532–554. 10.1080/09647775.2025.2467703

[ref31] Desvallées A Mairesse F : ΒασικέςΈννοιεςτηςMουσειολογίας *Βασικές Έννοιες της Μουσειολογίας* [Basic Concepts of Museology]. 2014. Reference Source

[ref133] Dossis MF Kazanidis I Valsamidis SI : Proposed open source framework for interactive IoT smart museums. *PCI '18: Proceedings of the 22nd Pan-Hellenic Conference on Informatics.* 2018; pp.294–299. 10.1145/3291533.3291586

[ref134] European Digital Humanism Initiative (EUDHIT) : The European Digital Humanism Initiative (EUDHIT) (Project No. 101212890). Horizon Europe, CORDIS. 2025. Accessed October 20, 2025. Reference Source

[ref32] Fernandes N Casteleiro-Pitrez J : Augmented Reality in Portuguese Museums: A Grounded Theory Study on the Museum Professionals’ Perspectives. *Multimodal Technologies and Interaction.* 2023;7(9):87–87. 10.3390/mti7090087

[ref135] Fletcher A Lee MJ : Current social media uses and evaluations in American museums. *Museum Management and Curatorship.* 2012;27(5):505–521. 10.1080/09647775.2012.738136

[ref136] Floridi L : *The Fourth Revolution: How the Infosphere is Reshaping Human Reality.* Oxford: OUP;2014.

[ref33] Frank SJ Frank AM : Complementing Connoisseurship with Artificial Intelligence. *Curator: The Museum Journal.* 2022;65(4):835–868. 10.1111/cura.12492

[ref34] Frost S Thomas MM Forbes AG : Art I Don’t Like: An Anti-Recommender System for Visual Art – MW19|Boston. 2019. Accessed July 22, 2024. Reference Source

[ref35] Fuentes-Moraleda L Lafuente-Ibañez C Alvarez NF : Willingness to Accept Social Robots in Museums: An Exploratory Factor Analysis according to Visitor Profile. *Library Hi Tech.* 2021;40:894–913. 10.1108/lht-07-2020-0180

[ref36] Gans HJ : *Popular Culture and High Culture.* New York: Basic Books;1974.

[ref37] Gao Z Braud T : VR-Driven Museum Opportunities: Digitized Archives in the Age of the Metaverse. *Artnodes.* 2023; (32). 10.7238/artnodes.v0i32.402462

[ref38] Gaugne R Barreau J-B Lécuyer F : EXtended Reality for Cultural Heritage. *Handbook of Cultural Heritage Analysis.* D’Amico S Venuti V editors. Springer, Cham;2022;1405–1437. 10.1007/978-3-030-60016-7_48 Reference Source

[ref39] Harrington M : Virtual Dioramas inside and Outside Museums with the AR Perpetual Garden. MW19: MuseWeb 2019. 2019. Accessed July 12, 2024. Reference Source

[ref41] Hooper-Greenhill E : Σκέψεις για τη μουσειακή εκπαίδευση και επικοινωνία στη μεταμοντέρνα εποχή [Reflections on Museum Education and Communication in the Postmodern Era]. *Αρχαιολογία και Τέχνες (Archeology and Arts).* 1999;72:47–49. Reference Source

[ref42] Hooper-Greenhill E : Changing Values in the Art Museum: Rethinking Communication and Learning. *International Journal of Heritage Studies.* 2000;6(1):9–31. 10.1080/135272500363715

[ref43] Hourdakis A Ieronimakis J : ‘Exhibiting’ Lifelong Learning in Museums: The Museum of Education/Xeniseum as a Space of Civic Understanding and Social Connectedness. *Academia.* 2020;106–131. 10.26220/aca.3219

[ref40] *How Wireless Data Loggers Streamline Museum Preservation Monitoring*|Onset’s HOBO Data Loggers:n.d.Accessed July 23, 2024. Reference Source Reference Source

[ref44] Huang M-H Rust RT : Artificial Intelligence in Service. *Journal of Service Research.* 2018;21(2):155–172. 10.1177/1094670517752459

[ref45] Hughes K Moscardo G : For Me or Not for Me? Exploring Young Adults’ Museum Representations. *Leisure Sciences.* 2019;41(6):516–534. 10.1080/01490400.2018.1550455

[ref46] Hutson J Hutson P : Museums and the Metaverse: Emerging Technologies to Promote Inclusivity and Engagement. *Application of Modern Trends in Museums.* 2023. 10.5772/intechopen.110044

[ref47] ICOM Approves a New Museum Definition: International Council of Museums. 2022. Accessed July 23, 2024. Reference Source

[ref48] Iio T Satake S Kanda T : Human-like Guide Robot That Proactively Explains Exhibits. *Int. J. Soc. Robot.* 2019;12(2):549–566. 10.1007/s12369-019-00587-y

[ref49] Interactive Holograms: Survivor Stories Experience: Illinois Holocaust Museum. n.d.Accessed July 23, 2024. Reference Source

[ref137] Islam RU Viviani M Colace S : Improving Visitor Experience in Smart Museums. In: 2025 IEEE Latin Conference on IoT (LCIoT). Proceedings of the 2025 IEEE Latin Conference on IoT (LCIoT).Fortaleza, Brazil;2025; pp.116–120. 11118616. 10.1109/LCIoT64881.2025

[ref138] Ismail MMR Nessim AA Fathy F : Factors affecting museum buildings and heritage spaces in terms of energy optimization and comfort. *Alexandria Engineering Journal.* 2024;15(5):103069–103094. 10.1016/j.asej.2024.103069

[ref139] Ismail N Mat Som AP Hanafiah MH : Profiling the Millennial Visitors of the Smart Museum and Satisfaction of Visit: A Case Study of the Borneo Culture Museum, Sarawak Malaysia. *Information Management and Business Review.* 2025;17(1(I)S):87–100. 10.22610/imbr.v17i1(I)S.4441

[ref140] Ivanov R : Advanced Visitor Profiling for Personalized Museum Experiences Using Telemetry-Driven Smart Badges. *Electronics.* 2024;13(20):3977. 10.3390/electronics13203977

[ref141] Ivanov R Velkova V : Analyzing Visitor Behavior to Enhance Personalized Experiences in Smart Museums: A Systematic Literature Review. *Computers.* 2025;14(5):191. Reference Source

[ref142] Jangra S Singh G Mantri A : Exploring the impact of virtual reality on museum experiences: visitor immersion and experience consequences. *Virtual Reality.* 2025;29:84. Reference Source

[ref50] Kasperiuniene J Tandzegolskiene I : Smart Learning Environments in a Contemporary Museum: A Case Study. *Journal of Education Culture and Society.* 2020;11(2):353–375. 10.15503/jecs2020.2.353.375

[ref51] Katz B : The Louvre’s First VR Experience Lets Visitors Get close to the ‘Mona Lisa. *Smithsonian Magazine.* 2019. Accessed July 22, 2024. Reference Source

[ref54] Kayukawa S Sato D Masayuki M : Enhancing Blind Visitor’s Autonomy in a Science Museum Using an Autonomous Navigation Robot.CHI ‘23: Proceedings of the 2023 CHI Conference on Human Factors in Computing Systems. 2023; 541: 1-14. 10.1145/3544548.3581220

[ref52] Khadraoui WR : Inclusivity Practices & the Real Role of Technology in Art Museums – MW19|Boston. 2019. 2019. Accessed July 23, 2024. Reference Source

[ref53] Khan MN Rahman HU Faisal M : An IoT-Enabled Information System for Smart Navigation in Museums. *Sensors.* 2021;22(1):312. 10.3390/s22010312 35009853 PMC8749525

[ref143] Kim J Kim SY : Multidimensional Analysis of Visitor Interaction With Museum Apps: Designing for Enhanced Museum Experiences. *Curator.* 2025;68:622–636. 10.1111/cura.12677

[ref55] Kirova V : *Value Co-Creation and Value Co-Destruction through Interactive Technology in Tourism: The Case of ‘La Cité Du Vin.* Bordeaux, France: Wine Museum;2020. *Current Issues in Tourism*, February, 1–14. 10.1080/13683500.2020.1732883

[ref56] Komarac T Došen ĐO : Understanding Virtual Museum Visits: Generation Z Experiences. *Museum Management and Curatorship.* 2023;39:357–376. October, 1–20. 10.1080/09647775.2023.2269129

[ref57] Korzun D Varfolomeyev A Yalovitsyna S : Semantic Infrastructure of a Smart Museum: Toward Making Cultural Heritage Knowledge Usable and Creatable by Visitors and Professionals. *Personal and Ubiquitous Computing.* 2016;21(2):345–354. 10.1007/s00779-016-0996-7

[ref144] Kou Y Chen M : Digitalization in Chinese museums: a policy evolution perspective. *Museum Management and Curatorship.* 2025;40:609–641. 10.1080/09647775.2025.2467704

[ref145] Kung CH Lin PS : A Study on the Application of Artificial Intelligence in Interactive Museum Displays and Visitor Perception. *International Journal of Organizational Innovation.* 2024;17(2):155–167. Reference Source

[ref58] Kyprianidou E Papadaki E : Το ψηφιακό μάρκετινγκ σε μουσεία τέχνης και οργανισμούς παραστατικών τεχνών[ Cultural Organizations’ Digital Marketing: Web 2.0 Applications in Art Museums and Performing Arts Organizations]. *Πολιτιστικές βιομηχανίες και Τεχνοπολιτισμός: Πρακτικές και προκλήσεις [Cultural Industries and Technoculture: Practices and Challenges].* Theodosiou A Papadaki E Athens: Nissos;2018.

[ref66] López-Martínez A Carrera Á Iglesias CA : Empowering Museum Experiences Applying Gamification Techniques Based on Linked Data and Smart Objects. *Applied Sciences.* 2020;10(16):5419. 10.3390/app10165419

[ref59] Lee H-K Park S Lee Y : A Proposal of Virtual Museum Metaverse Content for the MZ Generation. *Digital Creativity.* 2022;33:79–95. 10.1080/14626268.2022.2063903

[ref60] Lehmannová M : 224 YEARS of DEFINING the MUSEUM. 2020. Reference Source

[ref61] Leue MC Jung T Dieck D t : Google Glass Augmented Reality: Generic Learning Outcomes for Art Galleries. *Information and Communication Technologies in Tourism.* 2014;2015:463–476. 10.1007/978-3-319-14343-9_34

[ref146] Li J Zheng X Watanabe I : A systematic review of digital transformation technologies in museum exhibition. *Computers in Human Behavior.* 2024;161:108407. 10.1016/j.chb.2024.108407

[ref62] Liestøl G : Museums, Artefacts and Cultural Heritage Sites. *The Journal of Media Innovations.* 2021;7(1):19–28. 10.5617/jomi.8792

[ref63] Liu S Guo J : Smart Museum and Visitor Satisfaction. *Journal of Autonomous Intelligence.* 2023;7(3). 10.32629/jai.v7i3.1242

[ref64] London D : Fostering Deep Engagement and Enhanced Learning through Wonder, Creativity, and Play. MuseWeb20:MW2020. 2020. Reference Source

[ref65] Longo MC Faraci R : Next-Generation Museum: A Metaverse Journey into the Culture. *Sinergie Italian Journal of Management.* 2023;41(1):147–176. Reference Source

[ref67] Lu SE Moyle B Reid S : Technology and Museum Visitor Experiences: A Four Stage Model of Evolution. *Information Technology & Tourism.* 2023;25:151–174. 10.1007/s40558-023-00252-1

[ref68] Ludden J Russick J : Digital Transformation: It’s a Process and You Can Start Now. MuseWeb20:MW2020. 2020.

[ref69] Lupetti ML Germak C Giuliano L : Robots and Cultural Heritage: New Museum Experiences. *BCS Learning & Development.* 2015. 10.14236/ewic/eva2015.36

[ref70] Manna R Palumbo R : What Makes a Museum Attractive to Young People? Evidence from Italy. *International Journal of Tourism Research.* 2018;20(4):508–517. 10.1002/jtr.2200

[ref147] Mao J Cao W Yu C : Study on Virtual Archaeological Animation Design and User Interaction in Mediterranean Smart Museums Using Deep Learning. *Mediterranean Archaeology and Archaeometry.* 2025;25(1). Reference Source

[ref71] Margetis G Apostolakis KC Ntoa S : X-Reality Museums: Unifying the Virtual and Real World towards Realistic Virtual Museums. *Applied Sciences.* 2020;11(1):338. 10.3390/app11010338

[ref72] Marques D : *The Visitor Experience Using Augmented Reality on Mobile Devices in Museum Exhibitions.* FEUP - Faculdade de Engenharia da Universidade do Porto;2017. Thesis. Reference Source

[ref73] Mason M : The MIT Museum Glassware Prototype. *Journal on Computing and Cultural Heritage.* 2016;9(3):1–28. 10.1145/2872278

[ref74] Mexico City Declaration on Cultural Policies: Mexico City. 1982. Unesco. Reference Source

[ref75] Mighali V Del Fiore G Patrono L : Innovative IoT-Aware Services for a Smart Museum. WWW ‘15 Companion: Proceedings of the 24th International Conference on World Wide Web. 2015; 547-550 10.1145/2740908.2744711

[ref76] Ming X : Augmented Reality (AR) in Art Museums: Reconfiguring and Mediating the Museum Dynamics. *Essay.utwente.nl.* 2018. June 29, 2018. Reference Source

[ref77] Modliński A Fortuna P Rożnowski B : Robots Onboard? Investigating What Individual Predispositions and Attitudes Influence the Reactions of Museums’ Employees towards the Adoption of Social Robots. *Museum Management and Curatorship.* 2023;39:457–481. 10.1080/09647775.2023.2235678

[ref79] Morin E : *Κοινωνιολογία [Sociology]. Translated by* Dimitris Dimoulas . Athens: Ekdosis tou eikostou protou;1998.

[ref80] Mucchi L Milanesi M Becagli C : Blockchain Technologies for Museum Management. The Case of the Loan of Cultural Objects. *Current Issues in Tourism.* 2022;25(18):3042–3056. 10.1080/13683500.2022.2050358

[ref81] MuseumsQuartier Wien as an Interactive Work of Art: B2B Austria. 2020. Accessed July 22, 2024. Reference Source

[ref82] Neuhofer B Buhalis D Ladkin A : Experiences, Co-Creation and Technology: A Conceptual Approach to Enhance Tourism Experiences. 2013. Reference Source

[ref148] Nguyen VQ Nhat Anh N Thi Huyen L : Mapping visitor experiences at museums: Research trends through bibliometric analysis. *Science & Technology Development Journal - Economics - Law and Management.* 2025;9(4):6258–6272. Reference Source

[ref83] Nisiotis L Alboul L : Initial Evaluation of an Intelligent Virtual Museum Prototype Powered by AI, XR and Robots. De Paolis LT Arpaia P Bourdot P , editors. *Augmented Reality, Virtual Reality, and Computer Graphics. AVR 2021. Lecture Notes in Computer Science.* vol 12980: Springer, Cham;2021; pp.290–305. 10.1007/978-3-030-87595-4_21

[ref84] OECD/ICOM: Culture and Local Development: Maximising the Impact: A Guide for Local Governments, Communities and Museums. 2019 September. 10.1787/9a855be5-en Reference Source

[ref85] Olaz X Garcia R Ortiz A : An Interdisciplinary Design of an Interactive Cultural Heritage Visit for In-Situ, Mixed Reality and Affective Experiences. *Multimodal Technologies and Interaction.* 2022;6(7):59. 10.3390/mti6070059

[ref149] Ozdemir G Zonah S : Revolutionising Heritage Interpretation with Smart Technologies: A Blueprint for Sustainable Tourism. *Sustainability.* 2025;17(10):4330. 10.3390/su17104330

[ref86] Palombini A : Storytelling and Telling History. Towards a Grammar of Narratives for Cultural Heritage Dissemination in the Digital Era. *Journal of Cultural Heritage.* 2017;24(March):134–139. 10.1016/j.culher.2016.10.017

[ref87] Pan R Zhang L Yang J : A Systematic Review of Smart Learning Environments. *Lecture Notes in Educational Technology.* 2022;11–20. 10.1007/978-981-19-5967-7_3

[ref88] Papadaki E : The Semiotics of Cultural Organisations’ On-Line Branding: The Examples of the Metropolitan Opera of New York and the National Opera of Greece. *Semiotics and Visual Communication III: Branded. The Semiotics of Branding in Culture and Context.* Zantides E , editor. Newcastle: Cambridge Scholars Publishing;2019; pp.426–49.

[ref90] Paschalidis G : Η Συμβολή του Πολιτισμού στην Κοινωνική και Οικονομική Ανάπτυξη [The Contribution of Culture to Social and Economic Development]. *Οι διαστάσεις των πολιτιστικών φαινομένων [The Dimensions of Cultural Phenomena].* Paschalidis G Chabouri-Ioannidou A , editors. Patras: Hellenic Open University;2002b; pp.221–243.

[ref89] Paschalidis G : Μαζική Κουλτούρα και Υψηλή Τέχνη [Mass Culture and High Art]. *Οι διαστάσεις των πολιτιστικών φαινομένων [The Dimensions of Cultural Phenomena].* Paschalidis G Chabouri-Ioannidou A , editors. Patras: Hellenic Open University;2002a; pp.87–151.

[ref91] Pérez A : Smart Museums. Definition and Presentation of a Smart Management Model for Museums. *Tourism and Heritage Journal.* 2023;4(January):126–139. 10.1344/thj.2022.4.8

[ref150] Philippopoulos PI Drivas IC Tselikas ND : A Holistic Approach for Enhancing Museum Performance and Visitor Experience. *Sensors.* 2024;24(3):966. 10.3390/s24030966 38339683 PMC10856862

[ref92] Pine BJ Gilmore JH : The Experience Economy: Past, Present and Future. *Handbook on the Experience Economy.* 2013;21–44. 10.4337/9781781004227.00007

[ref151] Puspasari S Siradjuddin IA Rachmansyah : A Review of AI and IoT Implementation in a Museum’s Ecosystem: Benefits, Challenges, and a Novel Conceptual Model. *International Journal of Advanced Computer Science and Applications.* 2025;16(2). 10.14569/IJACSA.2025.0160271

[ref93] Rahimi BF : A Model for Sociocultural Interactions in Museums. *Museum Management and Curatorship.* 2014;29(2):174–187. 10.1080/09647775.2014.888821

[ref94] Recupero A Talamo A Triberti S : Bridging Museum Mission to Visitors’ Experience: Activity, Meanings, Interactions, Technology. *Frontiers in Psychology.* 2019;10(September). 10.3389/fpsyg.2019.02092 31551900 PMC6746986

[ref95] Refae S : Potential of Adapting Smart Cultural Model Related to Contemporary Art: The Case of Jeddah Open-Air Sculpture Museum. *Civil Engineering and Architecture.* 2022;10(3A):86–92. 10.13189/cea.2022.101311

[ref152] Rentschler R Radbourne J : Relationship Marketing in the Arts: The New Evoked Authenticity.In: Sargeant A Wymer W , editors. *The Routledge Companion to Nonprofit Marketing.* New York, NY: Routedge;2008; pp.241–252. Accessed October 22, 2025. 10.4324/9780203936023.ch14 Reference Source

[ref96] Rizvic S Mijatovic B Boskovic D : Workflow of Extended Reality Applications for Museum Exhibitions. *IEEE Xplore.* 2022. August 1, 2022. 10.1109/BalkanCom55633.2022.9900866

[ref97] Saggese A Vento M Vigilante V : MIVIABot: A Cognitive Robot for Smart Museum. *Lect. Notes Comput. Sci.* 2019;15–25. 10.1007/978-3-030-29888-3_2

[ref153] Sarakinos A Lembessis A : Color Holography for the Documentation and Dissemination of Cultural Heritage: OptoClones™ from Four Museums in Two Countries. *Journal of Imaging.* 2019;5(6):59. 10.3390/jimaging5060059 34460497 PMC8320957

[ref99] Scott B Salili-James A Smith V : Robot-In-The-Loop: Prototyping Robotic Digitisation at the Natural History Museum. *Biodiversity Information Science and Standards.* 2023;7(September). 10.3897/biss.7.112947

[ref98] Seeing Impressionism in 3D: Art Gallery of Ontario. 2019. Accessed July 22, 2024. Reference Source

[ref78] Shah MN Fathihin N Ghazali M : A Systematic Review on Digital Technology for Enhancing User Experience in Museums. *Communications in Computer and Information Science.* 2018;886:35–46. 10.1007/978-981-13-1628-9_4

[ref154] Shi Y Abdul Ghafar M Yahaya MF : Augmented Reality for Interactive Experiences in Museums: A Review. *Idealogy Journal.* 2024;9(2):104–116. 10.24191/idealogy.v9i2.551

[ref155] Shlyakhetko O Fedushko S Gregus M : Smart Exhibits: AI Integration in Modern Museums. *Procedia Computer Science.* 2025;257:754–761. 10.1016/j.procs.2025.03.097

[ref100] Sifaki E : Οπτική Eπικοινωνία και Tέχνες: Ένα Παράδειγμα Ανάλυσης Οπτικού Σχεδιασμού [Visual Communication and the Arts: An Example of Visual Design Analysis]. *Βίωμα και Bασισμένες στην Tέχνη Ποιοτικές Μέθοδοι Έρευνας [Experience and Art-Based Qualitative Research Methods], edited by Marios Pourkos.* Athens: Nisides;2015.

[ref101] Simone C Cerquetti M La Sala A : Museums in the Infosphere: Reshaping Value Creation. *Museum Management and Curatorship.* 2021;36(4):322–341. 10.1080/09647775.2021.1914140

[ref102] Siountri K Skondras E Vergados DD : Towards a Smart Museum Using BIM, IoT, Blockchain and Advanced Digital Technologies. *Proceedings of the 3rd International Conference on Vision, Image and Signal Processing.* 2019. August. 10.1145/3387168.3387196

[ref156] Slyusar M Nikitenko V Voronkova V : Digital humanism in the age of the Internet and artificial intelligence: Challenges, opportunities, and prospects for development. *European Journal of Management Issues.* 2024;10(5):344–352. 10.30525/2256-0742/2024-10-5-344-352

[ref103] Spachos P Plataniotis KN : BLE Beacons for Indoor Positioning at an Interactive IoT-Based Smart Museum. *IEEE Syst. J.* 2020;14:3483–3493. 10.1109/jsyst.2020.2969088

[ref104] Steingut RR Patall EA Fong CJ : Research Synthesis Methods. *Res. Synth. Methods.* 2022. 10.4324/9781138609877-ree55-1

[ref157] Sterling C : Museums after progress. *Museums & Social Issues.* 2024;18(1-2):1–6. Reference Source

[ref105] Summers K : Magical Machinery? What AI Can Do for Museums. *American Alliance of Museums.* 2019. May 3, 2019. Reference Source

[ref106] Tassis T : *Ψηφιακός Ανθρωπισμός: Εικονιστικό Υποκείμενο και Τεχνητή Νοημοσύνη [Digital Humanism: Iconistic Subject and Artificial Intelligence].* Athens: Armos Publications;2019.

[ref158] Thiel S : Managing AI Developing Strategic and Ethical Guidelines for Museums.In *AI in Museums. Reflections, Perspectives and Applications.* Thiel S Bernhardt J , editors, Edition Museum 74. Transcript: Bielefeld: Germany;2023; pp.83–98. 10.14361/9783839467107

[ref159] Tresnawati D Mulyani A Nugraha C : An Augmented Reality Technology in Indoor Navigation for Smart Museum Using Bluetooth Low Energy Communication. In: 2024 International Conference on ICT for Smart Society (ICISS). *IEEE Conference Proceedings.* 2024. 10.1109/ICISS62896.2024.10751467

[ref160] Trunfio M Jung T : Mixed reality experiences in museums: Exploring the impact of functional elements of the devices on visitors’ immersive experiences and post-experience behaviours. *Information & Management.* 2022;59(7-8): 103698. 10.1016/j.im.2022.103698

[ref107] Trunfio M Lucia MD Campana S : Innovating the Cultural Heritage Museum Service Model through Virtual Reality and Augmented Reality: The Effects on the Overall Visitor Experience and Satisfaction. *Journal of Cultural Heritage.* 2021;17(1):1–19. 10.1080/1743873x.2020.1850742

[ref108] UNESCO report: Museums around the World in the Face of COVID-19:2021. Reference Source

[ref109] United Nations: Refworld|Transforming Our World: The 2030 Agenda for Sustainable Development. *Refworld.* 2015; 2015. Reference Source

[ref111] V&Α: Sustainability: Victoria and Albert Museum. n.d. Reference Source

[ref110] Varitimiadis S Kotis K Skamagis A : Towards Implementing an AI Chatbot Platform for Museums. *International Conference on Cultural Informatics, Communication & Media Studies.* 2020;1(1). 10.12681/cicms.2732

[ref113] Victoria and Albert Museum Annual Report and Accounts 2021-2022:2022. Reference Source

[ref114] Victoria and Albert Museum Annual Report and Accounts 2022-2023:2023. Reference Source

[ref112] Victoria and Albert Museum Annual Report and Accounts 2020 to 2021: GOV.UK. 2022. Accessed July 22, 2024. Reference Source

[ref115] Wang B : Digital Design of Smart Museum Based on Artificial Intelligence. Edited by Sang-Bing Tsai. *Mobile Information Systems.* 2021;2021(December):1–13. 10.1155/2021/4894131

[ref161] Wang X : Co-design of a Voice-Driven Interactive Smart Guide for Museum Accessibility and Management. *Journal of Audiovisual Translation.* 2024;7(1):1–24. 10.47476/jat.v7i1.2024.267

[ref116] Williams R : *Resources of Hope: Culture, Democracy, Socialism.* London: Verso;1989.

[ref162] Xu J Pan Y : The Future Museum: Integrating Augmented Reality (AR) and Virtual-text with AI-enhanced Information Systems. Journal of Wireless Mobile Networks, Ubiquitous Computing, and Dependable Applications. 2024;15:373–394. Accessed November 2, 2025. 10.58346/JOWUA.2024.I3.025 Reference Source

[ref163] Xu Y Zhou Y : Research on the Innovation of Augmented Reality Technology in Museum User Experience Design. *Journal of Arts and Cultural Studies.* 2025;4(1):1–15. 10.23112/acs25061901

[ref117] Yang S : Storytelling and User Experience in the Cultural Metaverse. *Heliyon.* 2023;9(4):e14759. 10.1016/j.heliyon.2023.e14759 37035365 PMC10073831

[ref164] Yang X Huang D : Influence Mechanisms of Museums’ Technological Embodiment and Tourist Satisfaction. *SAGE Open.* 2025;15:15. 10.1177/21582440251330020

[ref118] YiFei L Othman MK : Investigating the Behavioural Intentions of Museum Visitors towards VR: A Systematic Literature Review. *Computers in Human Behavior.* 2024;155:108167–108167. 10.1016/j.chb.2024.108167

[ref165] Yu TC Huang MX Liu YH : A Systematic Review of Integrating Mixed Reality and Artificial Intelligence in Museums: Enhancing Visitor Experiences and Innovating Exhibit Design. Proceedings of the Hawaii International Conference on System Sciences. 2025:1529–1538. Accessed November 2, 2025. 10.24251/HICSS.2025.185

[ref119] Zachila K Kotis K Paparidis E : Facilitating Semantic Interoperability of Trustworthy IoT Entities in Cultural Spaces: The Smart Museum Ontology. *IoT.* 2021;2(4):741–760. 10.3390/iot2040037

[ref120] Zhang R(R) Rahman AA : Dive in the Flow Experience: Millennials’ Tech-Savvy, Satisfaction and Loyalty in the Smart Museum. *Current Issues in Tourism.* 2022;25(22):3694–3708. 10.1080/13683500.2022.2070459

[ref121] Zhang X Yang D Yow CH : Metaverse for Cultural Heritages. *Electronics.* 2022;11(22):3730. 10.3390/electronics11223730

[ref122] Zollo L Rialti R Marrucci A : How Do Museums Foster Loyalty in Tech-Savvy Visitors? The Role of Social Media and Digital Experience. *Current Issues in Tourism.* 2021;25(18):2991–3008. 10.1080/13683500.2021.1896487

