# Supplementary material for: How Smart Can Museums Be? The Role of Cutting-Edge Technologies in Making Modern Museums Smarter
Source: F1000Res. 2025 Nov 26;14:480. Originally published 2025 May 7. [Version 2] doi: 10.12688/f1000research.156212.2 (PMC12817164; doi:10.12688/f1000research.156212.2)
Supplement: Peer review history

# Reviewer report: Reviewer response for version 2

**DOI:** 10.5256/f1000research.191170.r405488

**Published:** 2026-01-16

**Authors:** Tiurida Lily Anita

**Affiliations:** 1 Universitas Bina Nusantara, Jakarta, Indonesia

While many factual statements are well supported, several issues require attention to strengthen scientific rigor: 

 1. Some broad or trend-based claims (e.g., “exponential progress since 2017,” museums being “more hesitant” than tourism) are not consistently backed by specific comparative or longitudinal citations.

 2. A few historical or technical assertions (e.g., dates related to the digital era) are presented as factual but lack explicit in-text references.

 3. Several references rely on non-peer-reviewed or weakly specified sources (e.g., general web pages, Scribd-hosted material, undated “Reference Source” entries), which weakens evidentiary strength when used to support factual claims.

 Overall Recommendation

 The article is conceptually strong, timely, and well-positioned within current museum and digital heritage scholarship. With moderate revisions—primarily related to citation robustness and clarity of scope—it can be considered scientifically sound and valuable as a foundational framework for future empirical and applied research on smart museums.

Is the review written in accessible language?

Partly

Are all factual statements correct and adequately supported by citations?

Partly

Are the conclusions drawn appropriate in the context of the current research literature?

Yes

Is the topic of the review discussed comprehensively in the context of the current literature?

Yes

Reviewer Expertise:

Service Management, Hospitality Management, and Hotel Management

I confirm that I have read this submission and believe that I have an appropriate level of expertise to confirm that it is of an acceptable scientific standard, however I have significant reservations, as outlined above.

# Reviewer report: Reviewer response for version 2

**DOI:** 10.5256/f1000research.191170.r436113

**Published:** 2025-12-30

**Authors:** Muhammad Nawaz Khan

**Affiliations:** 1 Gachon University, Gyeonggi-do, South Korea

The authors have incorporated all the suggested changes, now suitable for Indexing from my side.

Is the review written in accessible language?

Yes

Are all factual statements correct and adequately supported by citations?

Yes

Are the conclusions drawn appropriate in the context of the current research literature?

Yes

Is the topic of the review discussed comprehensively in the context of the current literature?

Yes

Reviewer Expertise:

Add further tabulation of data for easier analysis, and add visuall results for analysis, also add some latest research

I confirm that I have read this submission and believe that I have an appropriate level of expertise to confirm that it is of an acceptable scientific standard.

# Reviewer report: Reviewer response for version 1

**DOI:** 10.5256/f1000research.171492.r405492

**Published:** 2025-08-26

**Authors:** Muhammad Nawaz Khan

**Affiliations:** 1 Gachon University, Gyeonggi-do, South Korea

Despite extensive research on technology-mediated visitor experiences, existing studies often examine individual technologies in isolation, leaving a holistic understanding of the smart museum underexplored. To address this gap, this paper synthesizes insights from 83 articles and museum reports published between 2014 and 2024, focusing on six main technology categories—Extended Reality (XR), Artificial Intelligence (AI), Internet of Things (IoT), robots, fusion approaches, and emerging tools such as blockchain and holography. From this review, 15 defining characteristics of the 21st-century smart museum are identified, offering a cultural–technological framework that integrates innovation with visitor engagement. This study contributes to the literature by bridging fragmented research, redefining the museum experience in the digital era, and providing practical guidance for cultural managers, professionals, and designers in conceptualizing and implementing smart museums.

Is the review written in accessible language?

Yes

Are all factual statements correct and adequately supported by citations?

Yes

Are the conclusions drawn appropriate in the context of the current research literature?

Yes

Is the topic of the review discussed comprehensively in the context of the current literature?

Yes

Reviewer Expertise:

Add further tabulation of data for easier analysis, and add visuall results for analysis, also add some latest research

I confirm that I have read this submission and believe that I have an appropriate level of expertise to confirm that it is of an acceptable scientific standard, however I have significant reservations, as outlined above.

# Response

**Published:** 2025-11-09

**Authors:** Χάρις Αυλωνίτου

Dear Reviewer,

 We sincerely thank you for your highly positive and encouraging feedback on our manuscript. We greatly appreciate your recognition of the holistic approach to the smart museum concept, the effort to bridge fragmented research, and the provision of practical guidance. In light of your constructive suggestions for further enhancing the article's rigor, we proceeded with the following elaborations and updates:

 We extensively updated our sources, incorporating new academic publications —primarily 36 peer-reviewed journal articles from 2024-2025—alongside a few foundational theoretical or complementary texts (8 sources). These additions enrich the knowledge base and confirm the credibility of our proposed profile for the ideal smart museum, further substantiating the existing fifteen attributes. Critically, the most recent literature strongly reinforced the necessity of considering the cultural and theoretical implications of emerging technologies, especially AI, affirming the urgency and validity of the cultural lens through which the smart museum is examined.

 The findings from this updated corpus were fully integrated into the description of the 15 attributes (now strictly chronologically ordered in Table 1) and throughout the Discussion section, fostering a dynamic dialogue across the entire body of references.

 We also added seven new figures (pie, line, and bar/column charts, as well as heatmaps). This variety of visual aids enabled a unified, systematic, and detailed analysis of the bibliography, examining source type, thematic, and chronological distribution, as well as the correlation of literature with smart museum attributes and their temporal evolution. Equally importantly, a new conceptual diagram (Figure 8) was introduced to further clarify the model, illustrating the interaction and integrity of the core pillars and attributes as a single interdependent, puzzle-like whole. These visualizations enhanced transparency, accessibility, and the validity of the findings, while also improving the clarification of the conceptual model.

 From the upgraded analysis and tabulated synthesis, further observations emerged regarding the interrelation of specific attributes with technologies and among themselves, as well as the evolving focus of the museum’s identity, which have been integrated into the Findings and Discussion sections. The Methodology was also refined to document the full steps of data processing, analysis, and synthesis.

 Minor conceptual refinements were also applied to ensure consistency, including replacing broad terminology (e.g., ‘ICTs’ with ‘digital technologies’ and ‘features and properties’ with ‘attributes’) and refining select attributes with clarifying paired descriptors (e.g., visitor-centered– *interactive*, *inclusive*–democratizing, *strategic*–calculated). These adjustments enhance conceptual precision, capture the literature’s nuances, and distinguish closely related characteristics within the smart museum profile.

 Overall, these improvements have strengthened the evidential foundation and enhanced the visual presentation of the proposed model. We are sincerely grateful for your invaluable feedback, which directly contributed to these enhancements.

 Sincerely,

 Charis Avlonitou

# Reviewer report: Reviewer response for version 1

**DOI:** 10.5256/f1000research.171492.r384222

**Published:** 2025-05-26

**Authors:** James Hutson

**Affiliations:** 1 Department of Art History and Visual Culture, Lindenwood University, Saint Charles, USA

The article offers a comprehensive and nuanced review of the evolving concept of the smart museum, articulating its emergence at the intersection of digital innovation, cultural policy, and visitor-centered museology. The authors ground their discussion in a wide-ranging synthesis of 83 scholarly sources and institutional reports, mapping technological integration across domains such as extended reality, artificial intelligence, robotics, and the Internet of Things. What distinguishes this review is its sustained engagement with the cultural and theoretical implications of these technologies, incorporating frameworks such as constructivist learning, digital humanism, and participatory culture to contextualize the smart museum not merely as a technologically enhanced space, but as a socio-culturally responsive institution. This layered approach reflects the depth of current literature and renders the work particularly valuable for scholars and practitioners seeking to understand the broader institutional transformations underway.

 Factual assertions throughout the article are consistently substantiated by up-to-date citations, with references spanning peer-reviewed publications, global conference proceedings, and foundational texts in museology and sociology. The authors skillfully integrate historical perspectives—from postwar shifts in New Museology to the pedagogical reforms of the 1980s—with cutting-edge developments in museum informatics and immersive experience design. Notably, the profile of the smart museum is not treated monolithically; rather, it is deconstructed into fifteen interrelated features (e.g., hybridity, authenticity, anthropocentrism), each supported by evidence and interpreted within the context of contemporary museum practice. The inclusion of visual aids such as the Smart Museum Diagram (Figure 1, p. 6) and implementation tables (Tables 1–3, pp. 7–10) enhances the clarity and accessibility of the conceptual model while reinforcing the scholarly rigor of the review.

 The conclusions drawn are appropriately cautious and forward-looking, acknowledging both the promise and the potential pitfalls of digitizing cultural heritage. The authors do not position smart technologies as solutions in themselves but argue for their strategic, ethically-informed integration into museum missions and audience engagement strategies. In doing so, they emphasize the importance of maintaining authenticity, fostering participatory ecosystems, and resisting techno-determinism. Their recommendation to use the proposed fifteen-feature profile as a framework for future empirical studies and institutional benchmarking is both practical and scholarly.

Is the review written in accessible language?

Yes

Are all factual statements correct and adequately supported by citations?

Yes

Are the conclusions drawn appropriate in the context of the current research literature?

Yes

Is the topic of the review discussed comprehensively in the context of the current literature?

Yes

Reviewer Expertise:

Artificial Intelligence in Education and Cultural Heritage; Digital Humanities; Human-Centered Design; Museology and Smart Technologies; Immersive Learning Environments; Gamification; Neuroaesthetics; Ethical and Inclusive AI; Research Methodology and Epistemology in the Age of Generative Media

I confirm that I have read this submission and believe that I have an appropriate level of expertise to confirm that it is of an acceptable scientific standard.

# Response

**Published:** 2025-11-09

**Authors:** Χάρις Αυλωνίτου

Dear Reviewer,

 Thank you very much for your thoughtful and positive review of our manuscript. We greatly appreciate your recognition of the historical contextualization of the museum's transformation, the nuanced treatment of the smart museum concept, the comprehensive synthesis of sources, and the integration of cultural and theoretical frameworks. Your encouraging evaluation affirms the scholarly rigor we sought to achieve.

 We have made minor refinements to the manuscript, primarily to further systematize the conceptual model and reinforce the evidence base for the proposed fifteen-feature profile through updated literature, tables, and diagrams. These additions clarify and confirm the original analysis without altering its core framework or conclusions.

 We are grateful for your valuable time and insightful assessment.

 Sincerely,

 Charis Avlonitou
